# Turing complete neural computation based on synaptic plasticity

**DOI:** 10.1371/journal.pone.0223451

**Published:** 2019-10-16

**Authors:** Jérémie Cabessa

**Affiliations:** 1 Laboratory of Mathematical Economics and Applied Microeconomics (LEMMA), University Paris 2 – Panthéon-Assas, 75005 Paris, France; 2 Institute of Computer Science, Czech Academy of Sciences, 18207 Prague 8, Czech Republic; Polytechnical Universidad de Madrid, SPAIN

## Abstract

In neural computation, the essential information is generally encoded into the neurons via their spiking configurations, activation values or (attractor) dynamics. The synapses and their associated plasticity mechanisms are, by contrast, mainly used to process this information and implement the crucial learning features. Here, we propose a novel Turing complete paradigm of neural computation where the essential information is encoded into discrete synaptic states, and the updating of this information achieved via synaptic plasticity mechanisms. More specifically, we prove that any 2-counter machine—and hence any Turing machine—can be simulated by a rational-weighted recurrent neural network employing spike-timing-dependent plasticity (STDP) rules. The computational states and counter values of the machine are encoded into discrete synaptic strengths. The transitions between those synaptic weights are then achieved via STDP. These considerations show that a Turing complete synaptic-based paradigm of neural computation is theoretically possible and potentially exploitable. They support the idea that synapses are not only crucially involved in information processing and learning features, but also in the encoding of essential information. This approach represents a paradigm shift in the field of neural computation.

## Introduction

How does the brain compute? How do biological neural networks encode and process information? What are the computational capabilities of neural networks? Can neural networks implement abstract models of computation? Understanding the computational and dynamical capabilities of neural systems is a crucial issue with significant implications in computational and system neuroscience, artificial intelligence, machine learning, bio-inspired computing, robotics, but also theoretical computer science and philosophy.

In 1943, McCulloch and Pitts proposed the concept of an *artificial neural network (ANN)* as an interconnection of neuron-like logical units [1]. This computational model significantly contributed to the development of two research directions: (1) Neural Computation, which studies the processing and coding of information as well as as the computational capabilities of various kinds of artificial and biological neural models; (2) Machine Learning, which concerns the development and utilization of neural network algorithms in Artificial Intelligence (AI).

The proposed study lies within the first of these two approaches. In this context, the computational capabilities of diverse kinds of neural networks have been shown to range from the finite automaton degree [1–3] up to the Turing [4] or even to the super-Turing levels [5–7] (see [8] for a survey of complexity theoretic results). In short, *Boolean recurrent neural networks* are computationally equivalent fo finite state automata; *analog neural networks* with rational synaptic weights are Turing complete; and analog neural nets with real synaptic weights as well as evolving neural nets are capable of super-Turing capabilities (cf. Table 1). These theoretical results have later been improved, motivated by the possibility to implement finite state machines on electronic hardwares (see for instance [9–13]). Around the same time, the computational power of *spiking neural networks* (instead of sigmoidal ones) has also been extensively studied [14, 15]. More recently, the study of *P systems*—parallel abstract models of computation inspired from the membrane structure of biological cells—has become a highly active field of research [16–18].

**Table 1:** Computational power of various models of recurrent neural networks. FSA, TM and TM/poly(A) stand for finite state automata, Turing machines and Turing machines with polynomial advice (which are super-Turing), respectively. **REG**, **P** and **P/poly** are the complexity classes decided in polynomial time by these three models of computation. The results in the case of classical computation can be found in [1–7, 19–24]. Results in alternative infinite computational frameworks have also been obtained [25–35].

	Boolean Static	Sigmoid		
		Static	Bi-valued Evolving	Evolving
Q	FSA	TM	TM/poly(A)	TM/poly(A)
	**REG**	**P**	**P/poly**	**P/poly**
R	FSA	TM/poly(A)	TM/poly(A)	TM/poly(A)
	**REG**	**P/poly**	**P/poly**	**P/poly**

Concerning the second direction, Turing himself brilliantly anticipated the two concepts of *learning* and *training* that would later become central to machine learning [36]. These ideas were realized with the introduction of the *perceptron* [37], which gave rise to the algorithmic conception of learning [38–40]. Despite some early limitation issues [41], the development of artificial neural networks has steadily progressed since then. Nowadays, artificial neural networks represent a most powerful class of algorithms in machine learning, thanks to their highly efficient training capabilities. In particular, *deep learning* methods—multilayer neural networks that can learn in supervised and/or unsupervised manners—have achieved impressive results in numerous different areas (see [42] for a brilliant survey and the references therein).

These approaches share a common and certainly sensible conception of neural computation that could be qualified as a *neuron-based computational framework*. According to this conception, the essential information is encoded into the neurons, via their spiking configurations, activation values or (attractor) dynamics. The synapses and their associated plasticity mechanisms are, by contrast, essentially used to process this information and implement the crucial learning features. For instance, in the simulation of abstract machines by neural networks, the computational states of the machines are encoded into activation values or spiking patterns of neurons [8]. Similarly, in most if not all deep learning algorithms, the input, output and intermediate information is encoded into activation values of input, output and hidden (layers of) neurons, respectively [42]. But what if the synaptic states would also play a crucial role in the encoding of information? What if the role of the synapses would not only be confined to the processing of information and learning processes, as crucial as these features might be? In short, what about a *synaptic-based computational framework*?

In biology, the various mechanisms of *synaptic plasticity* provide “the basis for most models of learning, memory and development in neural circuits” [43]. *Spike-timing-dependent plasticity (STDP)* refers to the biological Hebbian-like learning process according to which the synapses’ strengths are adjusted based on the relative timings of the presynaptic and postsynaptic spikes [38, 44, 45]. It is widely believed that STDP “underlies several learning and information storage processes in the brain, as well as the development and refinement of neuronal circuits during brain development” (see [46] and the references therein). In particular, fundamental neuronal structures like *synfire chains* [47–51] (pools of successive layers of neurons strongly connected from one stratum to the next by excitatory connections), synfire rings [52] (looping synfire chains) and *polychronous groups* [53] (groups of neurons capable of generating time-locked reproducible spike-timing patterns), have all been observed to emerge in self-organizing neural networks employing various STDP mechanisms [52–55]. On another level, regarding STDP mechnisms, it has been shown that synapses might change their strengths by jumping between discrete mechanistic states, rather than by simply moving up and down in a continuum of efficacy [56].

Based on these considerations, we propose a novel Turing complete synaptic-based paradigm of neural computation. In this framework, the essential information is encoded into discrete synaptic states instead of neuronal spiking patterns, activation values or dynamics. The updating of this information is then achieved via synaptic plasticity mechanisms. More specifically, we prove that any 2-counter machine—and hence any Turing machine—can be simulated by a rational-weighted recurrent neural network subjected to STDP. The computational states and counter values of the machine are encoded into discrete synaptic strengths. The transitions between those synaptic weights are achieved via STDP. These results show that a Turing complete synaptic-based paradigm of computation is theoretically possible and potentially exploitable. They support the idea that synapses are not only crucially involved in information processing and learning features, but also in the encoding of essential information in the brain. This approach represents a paradigm shift in the field of neural computation.

The possible impacts of these results are both practical and theoretical. In the field of neuromorphic computing, our synaptic-based paradigm of neural computation might lead to the realization of novel analog neuronal computers implemented on VLSI technologies. Regarding AI, our approach might lead to the development of new machine learning algorithms. On a conceptual level, the study of neuro-inspired paradigms of abstract computation might improve the understanding of both biological and artificial intelligences. These aspects are discussed in the conclusion.

## Materials and methods

### Recurrent neural networks

A *rational-weighted recurrent neural network (RNN)* N consists of a synchronous network of neurons connected together in a general architecture. The network is composed of *M* input neurons ${\left(u_{i}\right)}_{i=1}^{M}$ and *N* internal neurons ${\left(x_{i}\right)}_{i=1}^{N}$. The dynamics of network N is computed as follows: given the activation values of the input neurons ${\left(u_{j}\left(t\right)\right)}_{j=1}^{M}$ and internal neurons ${\left(x_{j}\left(t\right)\right)}_{j=1}^{N}$ at time step *t*, the activation values of the internal neurons ${\left(x_{i}\left(t+1\right)\right)}_{i=1}^{N}$ at time step *t* + 1 are given by the following equations:

$$\begin{array}{c} x_{i}\left(t+1\right)=f(\sum_{j=1}^{N}a_{ij}\left(t\right)\cdot x_{j}\left(t\right)+\sum_{j=1}^{M}b_{ij}\left(t\right)\cdot u_{j}\left(t\right)+c_{i}\left(t\right)),\text{for}\;i=1,\cdots ,N \end{array}$$ (1)

where $a_{ij}\left(t\right),b_{ij}\left(t\right)\in Q$ are the rational *weights* of the synaptic connections from *x*_*j*_ to *x*_*i*_ and *u*_*j*_ to *x*_*i*_ at time *t*, respectively, $c_{i}\left(t\right)\in Q$ is the rational *bias* of cell *x*_*i*_ at time *t*, and *f* is either the *hard-threshold* activation function *θ* or the *linear sigmoid* activation function *σ* defined by

$$\begin{array}{c} \theta \left(x\right)=\{\begin{array}{cc} 0 & \text{if}\;x<1\\ 1 & \text{if}\;x\geq 1 \end{array}\;\;\sigma \left(x\right)=\{\begin{array}{cc} 0 & \text{if}\;x<0\\ x & \text{if}\;0\leq x\leq 1\\ 1 & \text{if}\;x>1. \end{array} \end{array}$$

A neuron is called *Boolean* or *analog* depending on whether its activation value is computed by the function *θ* or *σ*, respectively. Input neurons ${\left(u_{i}\right)}_{i=1}^{M}$ are all Boolean.

The *input state* and *internal state* of N at time *t* are the vectors

$$\begin{array}{cc} u(t) & ={\left(u_{1}\left(t\right),\ldots ,u_{M}\left(t\right)\right)}^{T}\in B^{M}\\ x(t) & ={\left(x_{1}\left(t\right),\ldots ,x_{N}\left(t\right)\right)}^{T}\in Q^{N} \end{array}$$

**Fig 1:**
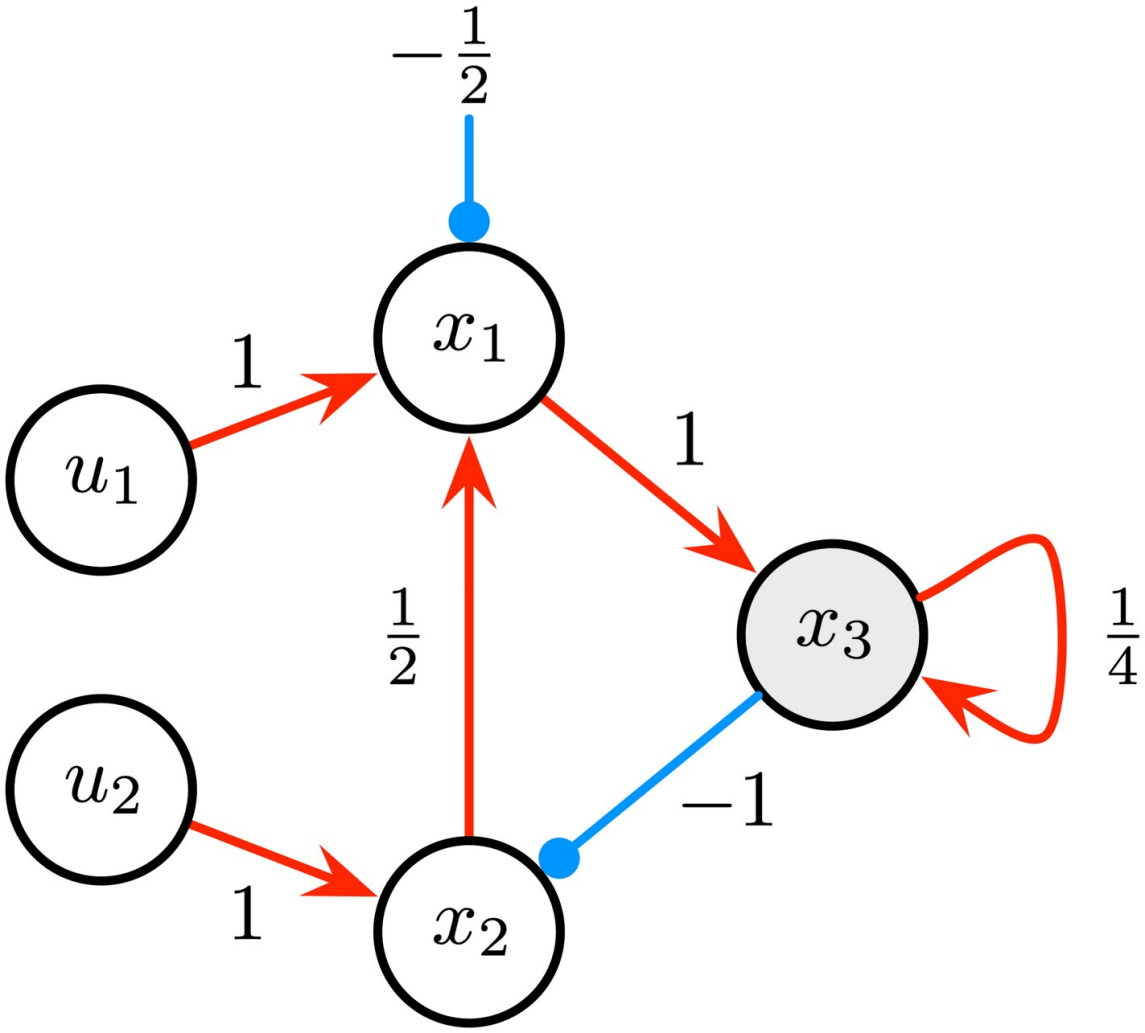
A recurrent neural network. The network contains two input cells *u*_1_, *u*_2_ and three internal cells *x*_1_, *x*_2_, *x*_3_. Excitatory and inhibitory connections are represented as red and blue arrows, respectively. Cells *u*_1_, *u*_2_, *x*_1_, *x*_2_ are Boolean (activation function *θ*) whereas *x*_3_ is analog (activation function *σ*). Over the Boolean input *u* = (1, 1)^*T*^ (1, 0)^*T*^ (0, 1)^*T*^, the network’s computation is $N\left(u\right)={\left(0,0,0\right)}^{T}{\left(0,1,0\right)}^{T}{\left(1,0,0\right)}^{T}{\left(0,1,1\right)}^{T}{\left(0,0,0.25\right)}^{T}{\left(0,0,0.625\right)}^{T}\ldots$.

For any Boolean input stream *u* = **u**(0)**u**(1)**u**(2) ⋯, the *computation* of N over input *u* is the sequence of internal states N(u)=x(0)x(1)x(2)…, where **x**(0) = **0** and the components of **x**(*t*) are given by Eq (1), for each *t* > 0. A simple recurrent neural network is illustrated in Fig 1.

**Fig 2:**
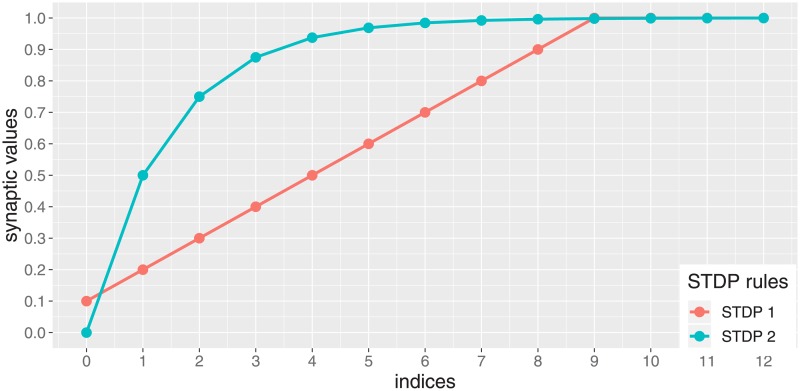
Synaptic weights enabled by to the two STDP rules. The red curve displays the finitely many possible synaptic weights enabled by the first STDP rule (Eq (2)), where *a*_*min*_ = 0.1, *a*_*max*_ = 1 and *η* = 0.1. These are the successive values of the sequence (0.1, 0.2, 0.3, 0.4, 0.5, 0.6, 0.7, 0.8, 0.9, 1.0). The blue curve displays the first elements of the infinitely many synaptic weights enabled by the second STDP rule (Eq (3)). These are the successive values of the sequence *β* = (0.0, 0.5, 0.75, 0.875, 0.9375, …).

A spike-timing dependent plasticity (STDP) rule modifies the synaptic weights *a*_*ij*_(*t*) according to the spiking patterns of the presynaptic and postsynaptic cells *x*_*j*_ and *x*_*i*_ [45]. Here, we consider two STDP rules. The first one is a classical generalized Hebbian rule [38]. It allows the synaptic weights to vary across finitely many values comprised between two bounds *a*_*min*_ and *a*_*max*_ (0 < *a*_*min*_ < *a*_*max*_ < 1). The rule is given as follows:

$$\begin{array}{c} a_{ij}\left(t+1\right)=\{\begin{array}{c} a_{min},\;\text{if}\;R\left(t+1\right)<a_{min}\\ a_{max},\;\text{if}\;R\left(t+1\right)>a_{max}\;\;\text{where}\\ R(t+1),\;\text{otherwise} \end{array} \end{array}$$ (2)

$$\begin{array}{c} R\left(t+1\right)≔a_{ij}\left(t\right)+\eta \cdot (\left\lfloor x_{i}\left(t+1\right)\right\rfloor \cdot \left\lfloor x_{j}\left(t\right)\right\rfloor -\left\lfloor x_{i}\left(t\right)\right\rfloor \cdot \left\lfloor x_{j}\left(t+1\right)\right\rfloor ) \end{array}$$

where ⌊*x*⌋ denotes the floor of *x* (the greatest integer less than or equal to *x*) and *η* > 0 is the *learning rate*. Accordingly, the synaptic weight *a*_*ij*_(*t*) is incremented (resp. decremented) by *η* at time *t* + 1 if the presynaptic cell *x*_*j*_ spikes 1 time step before (resp. after) the postsynaptic cell *x*_*i*_. The floor function is used to truncate the activation values of analog neurons to their integer part, if needed. The synaptic weights enabled by this rule is illustrated in Fig 2. In the sequel, this STDP rule will be used to encode the transitions between the finitely many computational states of the machine to be simulated.

The second rule is an adaptation to our context of a classical Hebbian rule. It allows the synaptic weights to vary across the infinitely many values of the sequence

$$\begin{array}{ccc} \beta ={\left(1-\frac{1}{2^{k}}\right)}_{k=0}^{\infty } & = & \left(0.0,0.5,0.75,0.875,0.9375,\ldots \right) \end{array}$$

The rule is given as follows:

$$\begin{array}{c} a_{ij}\left(t+1\right)=\{\begin{array}{cc} a_{ij}\left(t\right)+\frac{1}{2}\left(1-a_{ij}\left(t\right)\right) & \text{if}\;x_{i}\left(t+1\right)\cdot x_{j}\left(t\right)-x_{i}\left(t\right)\cdot x_{j}\left(t+1\right)=1\\ \text{max}\;(a_{ij}\left(t\right)-\left(1-a_{ij}\left(t\right)\right),0) & \text{if}\;x_{i}\left(t+1\right)\cdot x_{j}\left(t\right)-x_{i}\left(t\right)\cdot x_{j}\left(t+1\right)=-1\\ a_{ij}\left(t\right) & \text{if}\;x_{i}\left(t+1\right)\cdot x_{j}\left(t\right)-x_{i}\left(t\right)\cdot x_{j}\left(t+1\right)=0 \end{array} \end{array}$$ (3)

As for the previous one, the synaptic weight *a*_*ij*_(*t*) is incremented (resp. decremented) at time *t*+1 if the presynaptic cell *x*_*j*_ spikes 1 time step before (resp. after) the postsynaptic cell *x*_*i*_. But in this case, the synaptic weight varies across the infinitely many successive values of the sequence *β*. For instance, if $a_{ij}\left(t\right)=\frac{1}{2}+\frac{1}{4}+\frac{1}{8}=0.875$ is incremented (resp. decremented) by the STDP rule, then $a_{ij}\left(t+1\right)=\frac{1}{2}+\frac{1}{4}+\frac{1}{8}+\frac{1}{16}=0.9375$ (resp. $a_{ij}\left(t+1\right)=\frac{1}{2}+\frac{1}{4}=0.75$). Here, the floor functions are removed, since this rule will only be applied to synaptic connections between Boolean neurons. The synaptic weights enabled by this rule is illustrated in Fig 2. In the sequel, this STDP rule will be used to encode the variations among the infinitely many possible counter values of the machine to be simulated.

### Finite state automata

A deterministic *finite state automaton (FSA)* is a tuple $A=(Q,\Sigma ,\delta ,q_{0},F)$, where:

- *Q* = {*q*_0_, …, *q*_*n*−1_} is a finite set of *computational states*;
- Σ is an alphabet of *input symbols*;
- *δ*: *Q* × Σ → *Q* is a *transition function*;
- *q*_0_ ∈ *Q* is the *initial state*;
- *F* ⊆ *Q* is the set of *final states*.

Each transition *δ*(*q*, *a*) = *q*′ signifies that if the automaton is state *q* ∈ *Q* and reads input symbol *a* ∈ Σ, then it will move to state *q*′ ∈ *Q*. For any input *w* = *a*_0_*a*_1_ ⋯*a*_*p*_ ∈ Σ*, the *computation* of A over *w* is the finite sequence

$$\begin{array}{c} A\left(w\right)=(\left(q_{i_{0}},a_{0},q_{i_{1}}\right),\left(q_{i_{1}},a_{1},q_{i_{2}}\right),\ldots ,\left(q_{i_{p}},a_{p},q_{i_{p+1}}\right)) \end{array}$$

such that $q_{i_{0}}=q_{0}$ and $\delta \left(q_{i_{k}},a_{k}\right)=q_{i_{k+1}}$, for all *k* = 0, …, *p*. Such a computation is usually denoted as

$$\begin{array}{c} A\left(w\right):q_{0}\overset{a_{0}}{\to }q_{i_{1}}\overset{a_{1}}{\to }q_{i_{2}}\cdots q_{i_{p}}\overset{a_{p}}{\to }q_{i_{p+1}}. \end{array}$$

**Fig 3:**
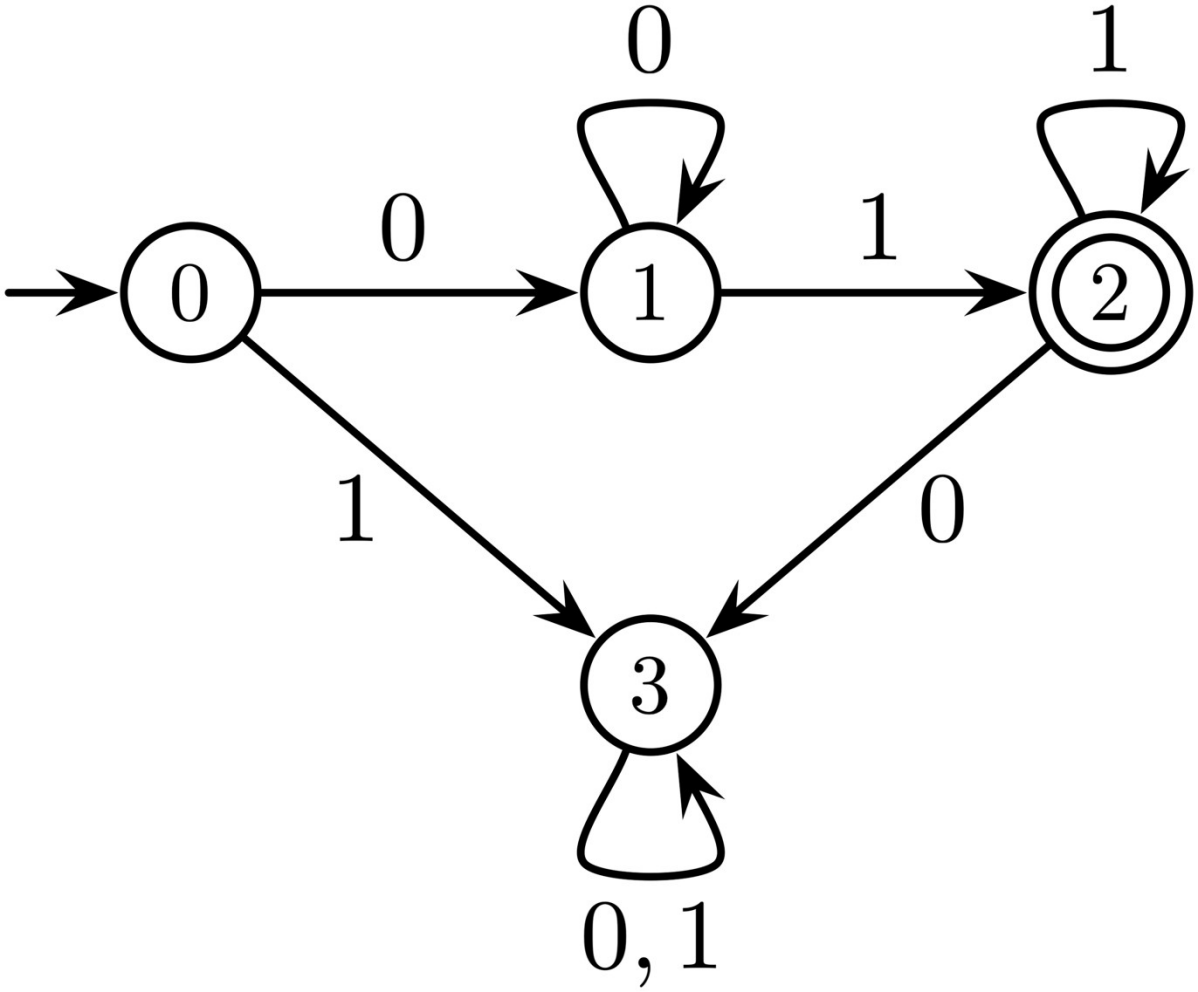
A finite state automaton. The nodes and edges of the graph represent the states and transitions of the automaton, respectively. Initial and final states are represented with an incoming arrow and a double-circle, respectively. An edge from state *q* to *q*′ labelled by *a* represents the transition relation *δ*(*q*, *a*) = *q*′. This automaton recognizes the language {0^*m*^1^*n*^: *m*, *n* > 0}, i.e., the sequences of bits beginning with a strictly positive number of 0’s and ending with a strictly positive number of 1’s.

Input *w* is said to be *accepted* (resp. *rejected*) by automaton A if the last state $q_{i_{p+1}}$ of computation A(w) belongs (resp. does not belong) to the set of final states *F*. The set of all inputs accepted by A is the *language* recognized by A. Finite state automata recognize the class of *regular* languages. A finite state automaton is generally represented as a directed graph, as illustrated in Fig 3.

### Counter machines

A *counter machine* is a finite state automaton provided with additional counters [57]. The counters are used to store integers. They can be pushed (incremented by 1), popped (decremented by 1) or kept unchanged. At each step, the machine determines its next computational state according to its current input symbol, computational state and counters’ states, i.e., if counters are zero or non-zero.

Formally, a deterministic *k*-*counter machine (CM)* is a tuple $C_{k}=\left(Q,\Sigma ,C,O,\delta ,q_{0},F\right)$, where:

- *Q* = {*q*_0_, …, *q*_*n*−1_} is a finite set of *computational states*;
- Σ is an alphabet of *input symbols* not containing the *empty symbol* *ϵ* (recall that the empty symbol satisfies *ϵw* = *wϵ* = *w*, for any string *w* ∈ Σ*);
- *C* = {⊥, ⊤} is the set of *counter states*, where ⊥, ⊤ represent the *zero* and *non-zero* states, respectively;
- N is the set of *counter values* (doesn’t need to be hold in the tuple $C_{k}$);
- *O* = {*push*, *pop*, −} is the set of *counter operations*;
- *δ*: *Q* × Σ ∪ {*ϵ*} × *C*^*k*^ → *Q* × *O*^*k*^ is a (partial) *transition function*;
- *q*_0_ ∈ *Q* is the *initial state*;
- *F* ⊆ *Q* is the set of *final states*.

The value and state of counter *j* are denoted by *c*_*j*_ and ${\bar{c}}_{j}$, respectively, for *j* = 1, …, *k*. (In the sequel, certain cells will also be denoted by *c*_*j*_’s and ${\bar{c}}_{j}$’s. The use of same notations to designate counter’s values or states and specific cells will be clear from the context.) The “bar function” ($c\mapsto \bar{c}$) retrieves the counter’s state from its value. It is naturally defined by ${\bar{c}}_{j}=⊥$ if *c*_*j*_ = 0 and ${\bar{c}}_{j}=⊤$ if *c*_*j*_ > 0. The value of counter *j* after application of operation *o*_*j*_ ∈ *O* is denoted by *o*_*j*_(*c*_*j*_). The counter operations influence their values in the following natural way:

- If *o*_*j*_ = *push*, then *o*_*j*_(*c*_*j*_) = *c*_*j*_ + 1;
- If *o*_*j*_ = *pop*, then *o*_*j*_(*c*_*j*_) = max(*c*_*j*_ − 1, 0);
- If *o*_*j*_ = −, then *o*_*j*_(*c*_*j*_) = *c*_*j*_.

Each transition $\delta \left(q,a,{\bar{c}}_{1},\ldots ,{\bar{c}}_{k}\right)=\left(q^{\prime },o_{1},\ldots ,o_{k}\right)$ signifies that if the machine is state *q* ∈ *Q*, reads the regular or empty input symbol *a* ∈ Σ ∪ {*ϵ*} and has its *k* counter being in states ${\bar{c}}_{1},\ldots ,{\bar{c}}_{k}\in C$, then it will move to state *q*′ ∈ *Q* and perform the *k* counter operations *o*_1_, …, *o*_*k*_ ∈ *O*. Depending on whether *a* ∈ Σ or *a* = *ϵ*, the corresponding transition is called a *regular transition* or an *ϵ*-*transition*, respectively. We assume that *δ* is a partial (rather than a total) function. Importantly, the determinism is expressed by the fact that the machine can never face a choice between either a regular or an *ϵ*-transition, i.e., for any *q* ∈ *Q*, any *a* ∈ Σ and any ${\bar{c}}_{1},\ldots ,{\bar{c}}_{k}\in C$, if $\delta (q,a,{\bar{c}}_{1},\ldots ,{\bar{c}}_{k})$ is defined, then $\delta (q,\epsilon ,{\bar{c}}_{1},\ldots ,{\bar{c}}_{k})$ is undefined [57].

For any input *w* = *a*_0_*a*_1_ ⋯ *a*_*p*_ ∈ Σ*, the *computation* of a k-counter machine $C_{k}$ over input *w* can be described as follows. For each successive input symbol *a*_*i*_ ∈ Σ, before trying to process *a*_*i*_, the machine first tests if an *ϵ*-transition is possible. If this is the case, it performs this transition. Otherwise, it tests if the regular transition associated with *a*_*i*_ is possible, and if so, performs it. The deterministic condition ensures that a regular and an *ϵ*-transition are never possible at the same time. When no more transition can be performed, the machine stops.

For any input *w* = *a*_0_*a*_1_ ⋯ *a*_*p*_ ∈ Σ*, the *computation* of $C_{k}$ over *w* is the unique finite or infinite sequence of states, symbols and counter values encountered by $C_{k}$ while reading the successive bits of *w* possibly interspersed with *ϵ* symbols. The formal definition involves the following notions.

An *instantaneous description* of $C_{k}$ is a tuple $\left(q,w,c_{1},\ldots ,c_{k}\right)\in Q\times \Sigma^{*}\times N^{k}$. For any empty or non-empty symbol *a*′ ∈ Σ ∪ {*ϵ*} and any *w* ∈ Σ*, the relation “⊢” over the set of instantaneous descriptions is defined as follows:

$$\begin{array}{ccc} \left(q,a^{\prime }w,c_{1},\ldots ,c_{k}\right)⊢\left(q^{\prime },w,c_{1}^{\prime },\ldots ,c_{k}^{\prime }\right) & \;\;\;\text{iff}\;\;\; & \delta \left(q,a^{\prime },{\bar{c}}_{1},\ldots ,{\bar{c}}_{k}\right)=\left(q^{\prime },o_{1},\ldots ,o_{k}\right)\\ & \;\;\;\text{and}\;\;\; & c_{1}^{\prime }=o_{1}\left(c_{1}\right),\ldots ,c_{k}^{\prime }=o_{k}\left(c_{k}\right) \end{array}$$

Note that depending on whether *a*′ = *ϵ* or *a*′ ∈ Σ, the relation “⊢” is determined by an *ϵ*-transition or a regular transition, respectively. (Note also that when *a*′ = *ϵ*, one has *a*′*w* = *ϵw* = *w*, and in this case, the relation “⊢” keeps *w* unchanged).

For any input *w* = *a*_0_*a*_1_ ⋯ *a*_*p*_ ∈ Σ*, the determinism of $C_{k}$ ensures that there is a unique finite or infinite sequence of instantaneous descriptions

$$\begin{array}{c} {\left((q_{n_{i}},w_{i},c_{1,i},\ldots ,c_{k,i})\right)}_{i=0}^{l},\;\;l\in N\cup \left\{\infty \right\} \end{array}$$

such that $\left(q_{n_{0}},w_{0},c_{1,0},\ldots ,c_{k,0}\right)=\left(q_{0},w,0,\ldots ,0\right)$ is the initial instantaneous description, and $\left(q_{n_{i}},w_{i},c_{1,i},\ldots ,c_{k,i}\right)⊢\left(q_{n_{i+1}},w_{i+1},c_{1,i+1},\ldots ,c_{k,i+1}\right)$, for all *i* < *l*. Then, the *computation* of $C_{k}$ over *w*, denoted by $C_{k}\left(w\right)$, is the finite or infinite sequence defined by

$$\begin{array}{c} C_{k}\left(w\right)={\left((q_{n_{i}},a_{i}^{\prime },c_{1,i},\ldots ,c_{k,i})\right)}_{i=0}^{l},\;\;l\in N\cup \left\{\infty \right\} \end{array}$$ (4)

where $a_{i}^{\prime }=\epsilon$ if *w*_*i*_ = *w*_*i*+1_ (case of an *ϵ*-transition), and $a_{i}^{\prime }$ is the first bit of *w*_*i*_ otherwise (case of a regular transition), for all *i* < *l*. Note that the computation over *w* can take longer than |*w*| = *p* + 1 steps, even be infinite, due to the use of *ϵ*-transitions. The input *w* ∈ Σ* is said to be *accepted* by $C_{k}$ if the computation of the machine over *w* is finite, consumes all letters of *w* and stops in a state of *F*, i.e., if $a_{l}^{\prime }=\epsilon$ and $q_{n_{l}}\in F$. It is *rejected* otherwise. The set of all inputs accepted by $C_{k}$ is the *language* recognized by $C_{k}$.

It is known that 1-counter machines are strictly more powerful than finite state automata, and *k*-counter machines are computationally equivalent to Turing machines (Turing complete), for any *k* ≥ 2 [57]. However, the class of *k*-counter machines that do not make use of *ϵ*-transitions is not Turing complete. For this reason, the simulation of *ϵ*-transitions by our neural networks will be essential towards the achievement of Turing-completeness.

**Fig 4:**
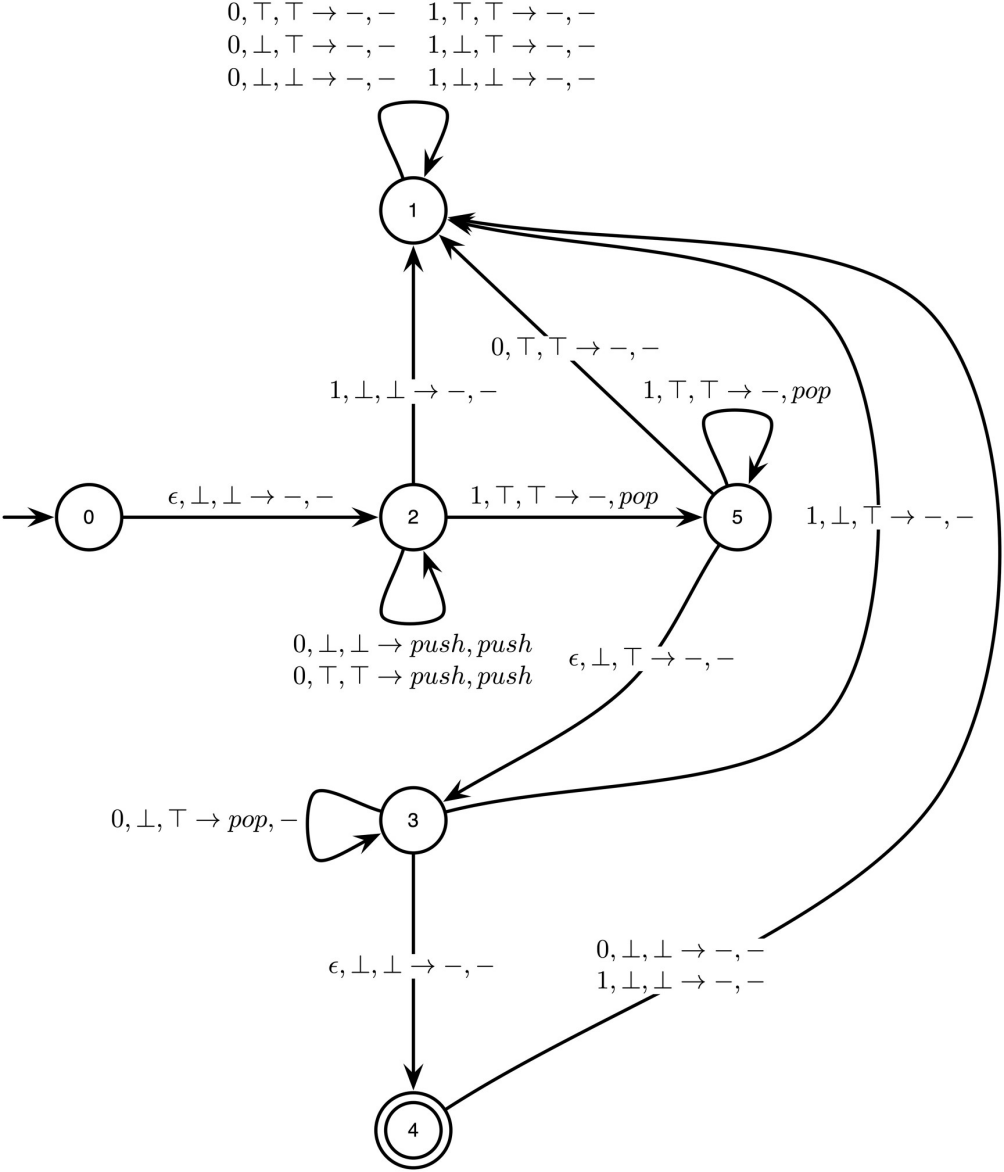
A 2-counter machine. The nodes and edges of the graph represent the states and transitions of the machine, respectively. An edge from *q* to *q*′ labelled by $a,{\bar{c}}_{1},{\bar{c}}_{2}\to o_{1},o_{2}$ represent the transition $\delta \left(q,a,{\bar{c}}_{1},{\bar{c}}_{2}\right)=\left(q^{\prime },o_{1},o_{2}\right)$. In other words, if the machine is in computational state *q*, reads input *a* and has counter states ${\bar{c}}_{1},{\bar{c}}_{2}$, then it will move to computational state *q*′ and performs counter operations *o*_1_, *o*_2_. This 2-counter machine recognizes the language {0^*n*^1^*n*^0^*n*^: *n* > 0}, i.e., the sequences of bits beginning with a strictly positive number of 0’s followed by the same number of 1’s and followed by the same number of 0’s again.

A *k*-counter machine can also be represented as a directed graph, as illustrated in Fig 4. The 2-counter machine of Fig 4 recognizes a language that is recursively enumerable but not context-free, i.e., it can be recognized by some Turing machine, yet by no pushdown automaton. Note that this 2-counter machine contains *ϵ*-transitions.

## Results

We show that *any* *k*-counter machine can be simulated by a recurrent neural network composed of Boolean and analog neurons, and using the two STDP rules described by Eqs (2) and (3). In this computational paradigm, the states and counter values of the machine are encoded into specific synaptic weights of the network. The transitions between those states and counter values are reflected by an evolution of the corresponding synaptic weights. Since 2-counter machines are computationally equivalent to Turing machines, these results show that the proposed STDP-based recurrent neural networks are Turing complete.

### Construction

We provide an algorithmic construction which takes the description of a *k*-counter machine $C_{k}$ as input and provides a recurrent neural network N that simulates $C_{k}$ as output. The network N is constructed by assembling several modules together: an *input encoding module*, an *input transmission module*, a *state module*, *k* *counter modules* and several *detection modules*. These modules are described in detail in the sequel. The global behaviour of N can be summarized as follows.

1. The computational state and *k* counter values of $C_{k}$ are encoded into specific synaptic weights belonging to the *state module* and *counter modules* of N, respectively.
2. At the beginning of the simulation, N receives its input stream via successive activations of its *input cells* belonging to the *input encoding module*. Meanwhile, this module encodes the whole input stream into a single rational number, and stores this number into the activation value of a sigmoid neuron.
3. Then, each time the so-called *tic* cell of the *input encoding module* is activated, N triggers the simulation of one computational step of $C_{k}$.
  - First, it attempts to simulate an *ϵ*-transition of $C_{k}$ by activating the cell *u*_*ϵ*_ of the *input transmission module*. If such a transition is possible in $C_{k}$, then N simulates it.
  - Otherwise, a signal is sent back the *input encoding module*. This module then retrieves the last input bit *a* stored in its memory, and attempts to simulate the regular transition of $C_{k}$ associated with *a* by activating the cell *u*_*a*_ of the *input transmission module*. If such a transition is possible in $C_{k}$, then N simulates it.
4. The network N simulates a transition of $C_{k}$ as follows: first, it retrieves the current computational state and *k* counter values of $C_{k}$ encoded into *k* + 1 synaptic weights by means of its *detection modules*. Based on this information, it sends specific signals to the *state module* and *counter modules*. These signals update specific synaptic weights of these modules in such a way to encode the new computational state and counter values of $C_{k}$.

**Fig 5:**
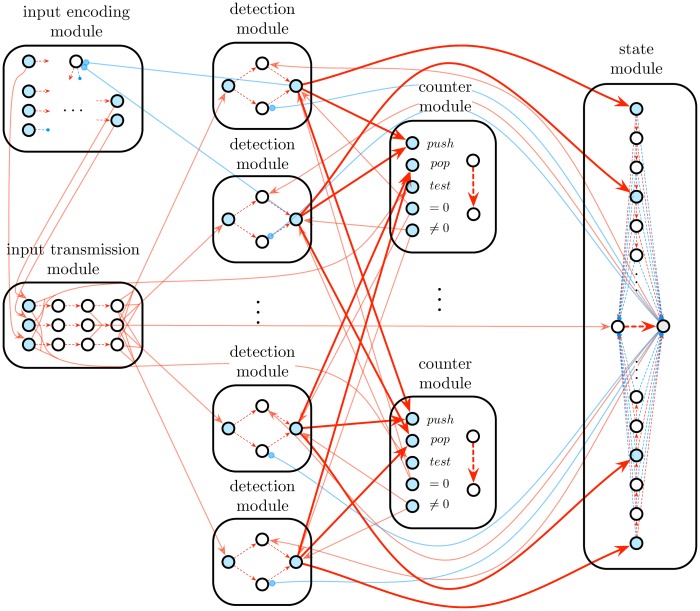
STDP-based recurrent neural network simulating a *k*-counter machine. The network is obtained by the construction given in Algorithm 1. It is composed of 1 input encoding module. 1 input transmission module, 1 state module, *k* counter modules, and at most |*Q*| ⋅ |Σ ∪ {*ϵ*}| ⋅ 2*k* = 6*nk* detection modules, all interconnected together in a precise way. According to this construction, the computational state and counter values of the machine are encoded into specific synaptic weights of the state and counter modules, respectively (red dashed arrow). The synaptic connections provoking changes in these specific weights are depicted in boldface.

The general architecture of N is illustrated in Fig 5. The general functionalities of the modules are summarized in Table 2. The following sections are devoted to the detailed description of the modules, as well as to the proof of correctness of the construction.

**Table 2:** Modules composing the STDP-based recurrent neural network that simulates a *k*-counter machine.

Module	Role
input encoding	Store the successive input bits into a “stack”. Implement a “tic mechanism” which triggers the simulation of one computational step of the machine.
input processing	Transmit the successive input bits to the network.
state	Encode the successive computational states of the machine into an evolving synaptic weight. Simulate the change in computational states of the machine throughout the computation.
counter	Encode the successive counter values of the machine into evolving synaptic weights. Simulate the change in the counter values of the machine throughout the computation.
detection	Retrieve the current computational and counter states of the machine. Use this information to simulate the next transition of the machine.

#### Stack encoding

In the sequel, each binary input stream will be piled up into a “binary stack”. In this way, the input stream can be stored by the network, and then processed bit by bit at successive time steps interspersed by constant intervals. The construction of the stack is achieved by “pushing” the successive incoming bits into it. The stack is encoded as a rational number stored in the activation value of one (or several) analog neurons. The pushing and popping stack operations can be simulated by simple analog neural circuits [4]. We now present these notions in detail.

A binary stack whose elements from top to bottom are *γ*_1_, *γ*_2_, …, *γ*_*p*_ ∈ {0, 1} is represented by the finite string *γ* = *γ*_1_*γ*_2_ ⋯ *γ*_*p*_ ∈ {0, 1}*. The stack *γ* whose top element has been popped is denoted by *pop*(*γ*) = *γ*_2_ ⋯ *γ*_*p*_, and the stack obtained by pushing element *α* ∈ {0, 1} into *γ* is denoted by *push*(*α*, *γ*) = *αγ*_1_ *γ*_2_ … *γ*_*p*_ (*α* is now the top element). For instance, if *γ* = 0110, then *pop*(*γ*) = 110, *push*(0, *γ*) = 00110 and *push*(1, *γ*) = 10110.

In our context, any stack *γ* = *γ*_1_*γ*_2_ ⋯ *γ*_*p*_ ∈ {0, 1}* is encoded by the rational number ${\bar{r}}_{\gamma }≔\sum_{i=1}^{n}\frac{2\gamma_{i}+1}{4^{i}}\in \left[0,1\right]$ [4]. Hence, the top element *γ*_1_ of *γ* can be retrieved by the operation $top\left(\gamma \right)=\sigma \left(4{\bar{r}}_{\gamma }-2\right)\in \left\{0,1\right\}$, where *σ* is the linear sigmoid function defined previously. The encodings of *push*(0, *γ*) and *push*(1, *γ*) are given by $\sigma (\frac{{\bar{r}}_{\gamma }}{4}+\frac{1}{4})$ and $\sigma (\frac{{\bar{r}}_{\gamma }}{4}+\frac{3}{4})$, respectively. The encoding of *pop*(*γ*) is given by $\sigma (4{\bar{r}}_{\gamma }-2top\left(\gamma \right)-1)$. As an illustration, the stack *γ* = 0110 is encoded by ${\bar{r}}_{\gamma }=\frac{1}{4}+\frac{3}{16}+\frac{3}{64}+\frac{1}{256}$. The top element of *γ* is $top\left(\gamma \right)=\sigma \left(1+\frac{3}{4}+\frac{3}{16}+\frac{1}{64}-2\right)=0$. The encodings of *push*(0, *γ*) and *push*(1, *γ*) are $\frac{1}{4}+\frac{1}{16}+\frac{3}{64}+\frac{3}{256}+\frac{1}{1024}$ and $\frac{3}{4}+\frac{1}{16}+\frac{3}{64}+\frac{3}{256}+\frac{1}{1024}$, which represents the stacks 00110 and 10110, respectively. The encoding of *pop*(*γ*) is $\sigma \left(1+\frac{3}{4}+\frac{3}{16}+\frac{1}{64}-2\cdot 0-1\right)=\frac{3}{4}+\frac{3}{16}+\frac{1}{64}$, which represents to the stack 110. These four operations can be implemented by simple neural circuits.

#### Input encoding module

**Fig 6:**
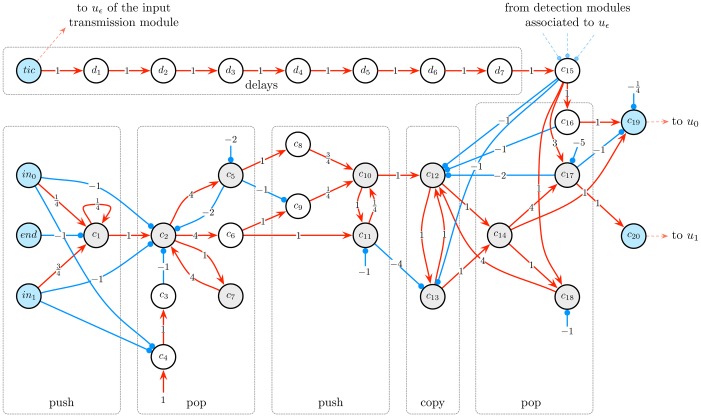
Input encoding module. This module piles up the successive incoming input bits into a stack and implement the “tic mechanism”, which triggers the simulation of one computational step of the counter machine. It is composed of 31 Boolean and analog cells (depicted in white/blue and grey, respectively) *in*_0_, *in*_1_, *end*, *tic*, *c*_1_, …, *c*_20_, *d*_1_, …, *d*_7_. First of all, at successive time steps, cell *in*_0_ or *in*_1_ spikes depending on whether input 0 or 1 is received. Then, cell *end* spikes to indicate that all input bits have been processed. Meanwhile, the successive bits are pushed into a stack *γ*′ whose encoding is hold by *c*_1_ (first ‘push’ circuit). After all bits have been pushed, *γ*′ contains all input bits in reverse order. Subsequently, *c*_2_, …, *c*_7_ pop every element of *γ*′ (first ‘pop’ circuit). Cell *c*_8_ or *c*_9_ spikes iff the popped element is a 0 or a 1, respectively. Afterwards, cells *c*_10_, *c*_11_ push these elements back into a new stack, in order to build the reversed stack *γ* (second ‘push’ circuit). The encoding of *γ* is transferred to and hold by *c*_12_ and *c*_13_ at alternating time steps (‘copy’ circuit), and then hold by *c*_14_ at every time step. After completion of this process, *γ* contains all input bits in the original order. Besides this, each time the *tic* cells spikes, it triggers the simulation of one computational step of the counter machine by the network. First, it attempts to simulatate an *ϵ*-transition by activating cell *u*_*ϵ*_ of the next module. If this simulation step fails, cell *c*_15_ is activated after some delay (‘delays’ circuit), which represents a signal telling that the top element of stack *γ*, instead of *ϵ*, has to be given as next input symbol. In this case, *c*_14_, *c*_16_, *c*_17_, *c*_18_ pop *γ* (second ‘pop’ circuit) and transmit its top element, 0 or 1, to cell *c*_19_ or *c*_20_, respectively. Cell *c*_19_ or *c*_20_ then activates cell *u*_0_ or *u*_1_ of the next module, respectively, triggering the simulation of a regular transition.

The *input encoding module* is used for two purposes: pile up the successive input bits into a stack, and implement a “tic mechanism” which triggers the simulation of one computational step of the counter machine by the network. These two processes are described in detail below. This module (the most intricate one) has been designed on the basis of the previous considerations about stack encoding, involving neural circuits that implement the “pop”, “top” and “pop” operations. It is composed of 31 cells *in*_0_, *in*_1_, *end*, *tic*, *c*_1_, …, *c*_20_, *d*_1_, …, *d*_7_, some of which being Boolean and others analog, as illustrated in Fig 6. It is connected to the *input transmission module* and the *detection modules* described below.

The three Boolean cells *in*_0_, *in*_1_ and *end* are input cells of the network. They are used to transmit the successive inputs bits to the network. The transmission of input 0 or 1 is represented by a spike of cell *in*_0_ or *in*_1_, respectively. At the end of the input stream, cell *end* spikes to indicate that all inputs have been processed.

**Fig 7:**
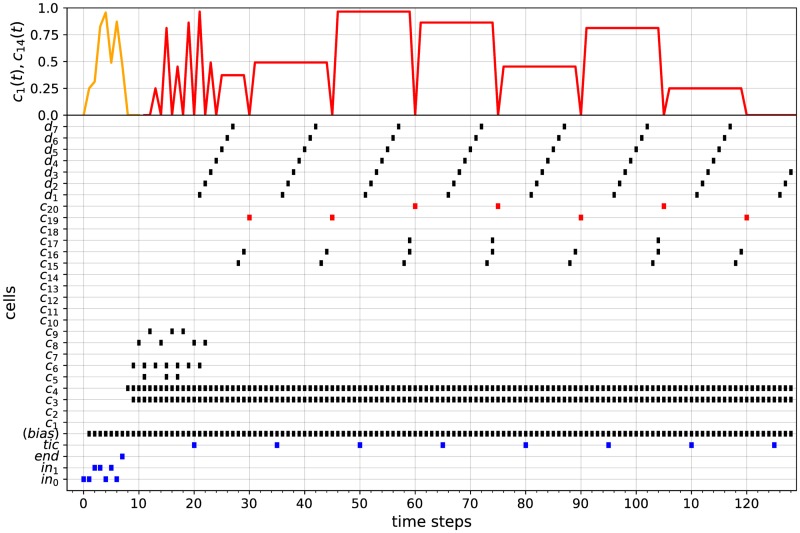
Example of activity of the input encoding module. The lower graph is a raster plot displaying the cells’ activities. Activation values between 0 and 1 (of sigmoid neurons) are not represented, only spikes are. In this simulation, the input stream 001101 and the “end of input” signal are transmitted via cells *in*_0_, *in*_1_, *end* at successive time steps 0, 1, 2, …, 7 (blue pattern). The successive input bits are first piled up in reverse order into a stack *γ*′ whose encoding is stored as the activation value of *c*_1_, and then piled up again in the right order into a stack *γ* whose encoding is stored as the activation value of *c*_14_. The activation values of *c*_1_ and *c*_14_ over time are represented by the orange and red curves in the upper graph, respectively. Then, the *tic* cell spikes every 15 time steps from *t* = 20 onwards (blue pattern). Each such spike triggers the sub-circuit that pops stack *γ* and outputs its top element, 0 or 1, by activating cell *c*_19_ or *c*_20_ 10 time steps later, respectively. We see that the successive input bits, namely 0, 0, 1, 1, 0, 1, 0 (blue pattern), are correctly output by cells *c*_19_ or *c*_20_ (red pattern).

The activity of this module, illustrated in Fig 7, can be described as follows. Suppose that the input stream *a*_1_ ⋯ *a*_*p*_ is transmitted to the network. While the bits *a*_1_, …, *a*_*p*_ are being received, the module builds the stack *γ* = *a*_1_ ⋯ *a*_*p*_, and stores its encoding ${\bar{r}}_{\gamma }$ into the activation values of an analog neuron. To achieve this, the module first pushes every incoming input *a*_*i*_ into a stack *γ*′ (first ‘push’ circuit in Fig 6). Since pushed elements are by definition added on the top of the stack, *γ*′ consists of elements *a*_1_, …, *a*_*p*_ in reverse order, i.e., *γ*′ = *a*_*p*_ ⋯ *a*_1_. The encoding ${\bar{r}}_{\gamma^{\prime }}$ of stack *γ*′ is stored in cell *c*_1_. Then, the module pops the elements of *γ*′ from top to bottom (first ‘pop’ circuit in Fig 6), and pushed them into another stack *γ* (second ‘push’ circuit in Fig 6). After completion of this process, *γ* consists of elements *a*_1_, …, *a*_*p*_ in the right order, i.e., *γ* = *a*_1_ ⋯ *a*_*p*_. The encoding ${\bar{r}}_{\gamma }$ of stack *γ* is stored in cell *c*_14_.

The Boolean cell *tic* is also an input cell. Each activation this cell triggers the simulation of one computational step of the counter machine by the network. When the *tic* cell spikes, it sends a signal to cell *u*_*ϵ*_ of the next input transmission module. The activation of *u*_*ϵ*_ attempts to launch the simulation of an *ϵ*-transition of the machine. If, according to the current computational and counter states of the machine, an *ϵ*-transition is possible, then the network simulates it via its other modules, and at the same time, sends an inhibitory signal to *c*_15_. Otherwise, after some delay (‘delays’ circuit in Fig 6), cell *c*_15_ is activated. This cell triggers a sub-circuit that pops the current stack *γ* (second ‘pop’ circuit in Fig 6) and transmits its top element *a* ∈ {0, 1} to cell *u*_*a*_ of the next input transmission module. Then, the activation of *u*_*a*_ launches the simulation of a regular transition of the machine associated with input symbol *a*, via the other modules of the network.

The module is composed of several sub-circuits that implement the *top*(), *push*() and *pop*() operations described previously, as shown in Fig 6. An input encoding module is denoted as *input*_*encoding*_*module*().

#### Input transmission module

**Fig 8:**
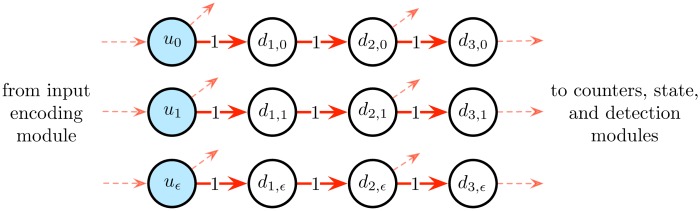
Input transmission module. This module transmits the successive inputs bits to the network. It is composed of three Boolean input cells *u*_0_, *u*_1_, *u*_*ϵ*_ (in blue) followed by 3 layers of Boolean delay cells connected in a parallel way via excitatory connections of weights 1. The activation of cells *u*_0_, *u*_1_ or *u*_*ϵ*_ simulates the reading of input symbols 0, 1 or *ϵ* by the counter machine, respectively.

The *input transmission module* is used to transmit to the network the successive input bits sent by the previous input encoding module. The module simply consists of 3 Boolean input cells *u*_0_, *u*_1_, *u*_*ϵ*_ followed by 3 layers of Boolean delay cells, as illustrated in Fig 8. It is connected to the *input encoding module* described above, and to the *state module*, *counter modules* and *detection modules* described below. The activation of cell *u*_0_, *u*_1_ or *u*_*ϵ*_ simulates the reading of input symbol 0, 1 or *ϵ* by the counter machine, respectively. Each time such a cell is activated, the information propagates along the delay cells of the corresponding row. An input transmission module is denoted as *input*_*transmission*_*module*().

#### State module

In our model, the successive computational states of the counter machine are encoded as rational numbers, and stored as successive weights of a designated synapse *w*_*s*_(*t*) (subscript *s* refers to ‘state’). More precisely, the fact that the machine is in state *q*_*k*_ is encoded by the rational weight *w*_*s*_(*t*) = *a*_*min*_ + *k* ⋅ *η*, for *k* = 0, …, *n* − 1, where *a*_*min*_ and *η* are parameters of the STDP rule given by Eq (2). Hence, the change in computational state of the machine is simulated by incrementing or decrementing *w*_*s*_(*t*) in a controlled manner. This process is achieved by letting *w*_*s*_(*t*) be subjected to the STDP rule of Eq (2), and by triggering specific spiking patterns of the presynaptic and postsynaptic cells of *w*_*s*_(*t*).

**Fig 9:**
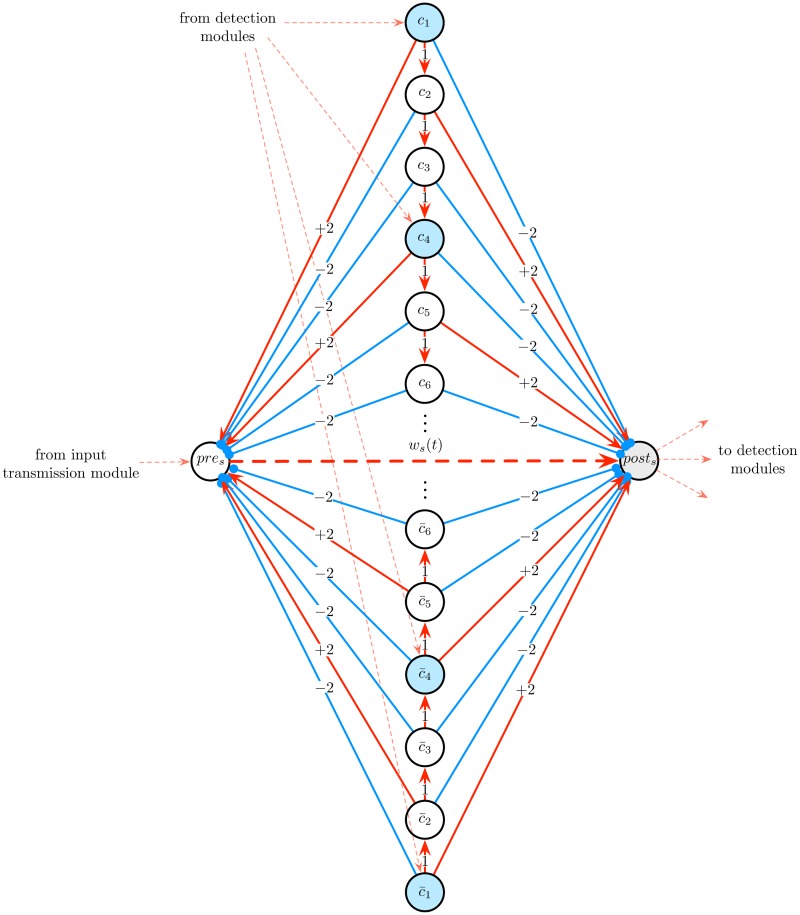
State module. This module is used to simulate the successive computational states of the counter machine. It is composed of a Boolean cell *pre*_*s*_ connected to an analog cell *post*_*s*_ via a synaptic connection of weight *w*_*s*_(*t*) (dashed red arrow) subjected to the first STDP rule given by Eq (2), as well as of 6*n* Boolean cells *c*_1_, …, *c*_3(*n*−1)_ and ${\bar{c}}_{1},\ldots ,{\bar{c}}_{3(n-1)}$. The latter cells project onto *pre*_*s*_ and *post*_*s*_ via excitatory and inhibitory synapses. To increment (resp. decrement) the value of *w*_*s*_(*t*) by (*n* − 1 − *k*) ⋅ *η* (where *η* is the learning rate of the STDP rule of Eq (2)), it suffices to activate the blue cell *c*_3*k*+1_ (resp. cell ${\bar{c}}_{3k+1}$), where 0 ≤ *k* ≤ *n* − 2.

The *state module* is designed to implement these features. It is composed of a Boolean presynaptic cell *pre*_*s*_ connected to an analog postsynaptic cell *post*_*s*_ by a synapse of weight *w*_*s*_(*t*), as well as of 6(*n* − 1) Boolean cells *c*_1_, …, *c*_3(*n* − 1)_ and ${\bar{c}}_{1},\ldots ,{\bar{c}}_{3(n-1)}$ (for some *n* to be specified), as illustrated in Fig 9. The synaptic weight *w*_*s*_(*t*) is subjected to the STDP rule of Eq (2), and has an initial value of *w*_*s*_(0) = *a*_*min*_. The architecture of the module ensures that the activation of cell *c*_3*k*+1_ or ${\bar{c}}_{3k+1}$ triggers successive specific spiking patterns of *pre*_*s*_ and *post*_*s*_ which, according to STDP (Eq (2)), increments or decrements *w*_*s*_(*t*) by (*n* − 1 − *k*) ⋅ *η*, for any 0 ≤ *k* ≤ *n* − 2, respectively (for instance, if *k* = 0, then *w*_*s*_(*t*) is incremented or decremented by (*n* − 1) ⋅ *η*, whereas if *k* = *n* − 2, then *w*_*s*_(*t*) is only incremented or decremented by 1 ⋅ *η*). The module is linked to the *input transmission module* described above and to the *detection modules* described below.

**Fig 10:**
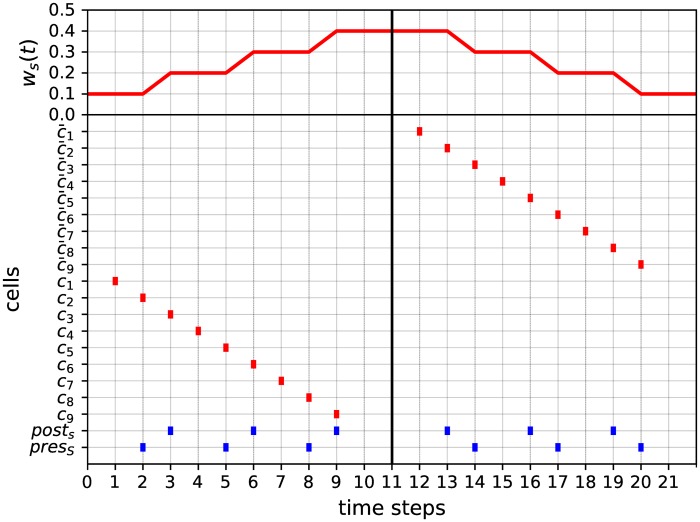
Example of activity of the state module. The lower graph is a raster plot displaying the cells’ activities. When cell *c*_1_ (resp. ${\bar{c}}_{1}$) spikes, it launches a chain of activations of the next cells *c*_2_, …, *c*_9_ (resp. ${\bar{c}}_{2},\ldots ,{\bar{c}}_{9}$). These activations (red events) induce spiking patterns of the cells *pre*_*s*_ and *post*_*s*_ (blue events), which thanks to the STDP rule of Eq (2), increment (resp. decrement) the synaptic weight *w*_*s*_(*t*) by steps of *η* (*η* = 0.1 here). The value of *w*_*s*_(*t*) over time is represented in the upper plot (red curve).

The activity of this module, illustrated in Fig 10, can be described as follows. Suppose that at time step *t*, one has *w*_*s*_(*t*) = *v* and one wishes to increment (resp. decrement) *w*_*s*_(*t*) by (*n* − 1 − *k*) ⋅ *η*, where *η* is the learning rate of the STDP rule of Eq (2) and 0 ≤ *k* ≤ *n* − 2. To achieve this, we activate the cell *c*_3*k*+1_ (resp. cell ${\bar{c}}_{3k+1}$) (a blue cell of Fig 9). The activation of *c*_3*k*+1_ (resp. cell ${\bar{c}}_{3k+1}$) launches a chain of activations of the next cells (red events in Fig 10), which, according to the connectivity of the module, induces *k* successive pairs of spikes of *pre*_*s*_ followed by *post*_*s*_ (resp. *post*_*s*_ followed by *pre*_*s*_) (blue events in Fig 10). Thanks to the STDP rule of Eq (2), these spiking patterns increment (resp. decrement) *k* times the value of *w*_*s*_(*t*) by an amount of *η*. A state module with 6(*n* − 1) + 2 cells is denoted as *state*_*module*(*n* − 1).

#### Counter module

In our model, the successive counter values of the machine are encoded as rational numbers and stored as successive weights of designated synapses $w_{c_{j}}\left(t\right)$, for *j* = 1, …, *k* (subscript *c*_*j*_ refers to ‘counter *j*’). More precisely, the fact that counter *j* has a value of *n* ≥ 0 at time *t* is encoded by the synaptic weight $w_{c_{j}}\left(t\right)$ having the rational value $r_{n}≔\sum_{i=1}^{n}\frac{1}{2^{i}}$ (with the convention that *r*_0_ ≔ 0). Then, the “push” (incrementing the counter by 1) and “pop” (decrementing the counter by 1) operations are simulated by incrementing or decrementing $w_{c_{j}}\left(t\right)$ appropriately.

**Fig 11:**
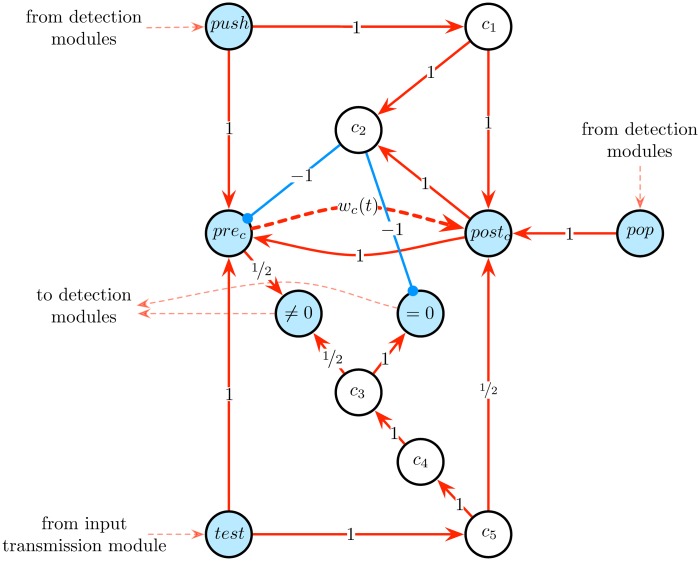
Counter module. This module is used to simulate one counter of a *k*-counter machine. It is composed of 12 Boolean cells: *push*, *pop*, *test*, = 0, ≠ 0, *pre*_*c*_, *post*_*c*_ (in blue), *c*_1_, *c*_2_, *c*_3_, *c*_4_, *c*_5_. The presynaptic and postsynaptic cells *pre*_*c*_ and *post*_*c*_ are connected by a synapse of weight *w*_*c*_(*t*) (dashed red arrow) subjected to the second STDP rule given by Eq (3). The activation of the *push* or *pop* cell increments or decrements the value of *w*_*c*_(*t*), respectively. The activation of the *test* cell results in the activation of the cell ‘= 0’ or ‘≠ 0’, depending on whether *w*(*t*) = 0 or *w*(*t*) ≠ 0, respectively.

The *k* *counter modules* are designed to implement these features. Each counter module is composed of 12 Boolean cells *push*, *pop*, *test*, = 0, ≠ 0, *pre*_*c*_, *post*_*c*_, *c*_1_, *c*_2_, *c*_3_, *c*_4_, *c*_5_, as illustrated in Fig 11. The presynaptic and postsynaptic cells *pre*_*c*_ and *post*_*c*_ are connected by a synapse of weight *w*_*c*_(*t*) subjected to the second STDP rule given by Eq (3) and having an initial value of *w*_*c*_(0) = 0. Accordingly, the values of *w*_*c*_(*t*) may vary across the elements of the infinite sequence $\beta ={\left(1-\frac{1}{2^{k}}\right)}_{k=0}^{\infty }=\left(0,0.5,0.75,0.875,0.9375,\ldots \right)$. The module is connected to the *input transmission module* described above and to *detection modules* described below.

**Fig 12:**
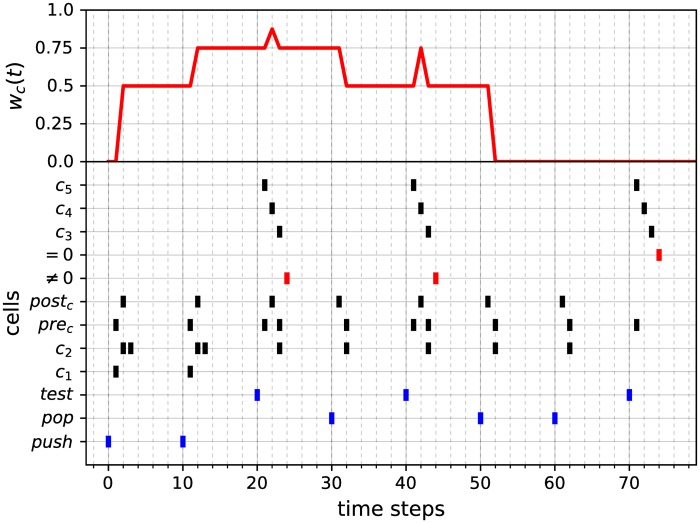
Example of activity of the counter module. The lower graph is a raster plot displaying the cells’ activities. Cells *push*, *push*, *test*, *pop*, *test*, *pop*, *pop*, *test* are activated at successive time steps 0, 10, 20, 30, 40, 50, 60, 70 (blue pattern). The upper curve shows the fluctuation of the synaptic weight *w*_*c*_(*t*), which encodes the change in the counter value over time. Note that the activations of the *push* and *pop* cells correctly increment and decrement the value of *w*_*c*_(*t*), respectively. At time 60, when *w*_*c*_(*t*) = 0 (counter is zero), the pop signal has no more effect on its value. Moreover, test queries are performed at times 20, 40 and 70 and their answers given by the activities of cells ‘= 0’ and ‘≠ 0’ (red pattern) at time 24, 44 and 74, respectively. Note that cells ‘= 0’ and ‘≠ 0’ provide correct answers to whether the value of *w*_*c*_(*t*) is 0 or not. Finally, note that whenever *w*_*c*_(*t*) ≠ 0, each testing procedure induces a fluctuation of *w*_*c*_(*t*) (peaks of the red curve), without finally modifying its initial value.

The activity of this module, illustrated in Fig 12, can be described as follows. Each activation of the *push* (resp. *pop*) cell (blue events in Fig 12) propagates into the circuit and results 2 time steps later in successive spikes of the *pre*_*c*_ and *post*_*c*_ cells (resp. *post*_*c*_ and *pre*_*c*_ cells), which, thanks to the STDP rule of Eq (3), increment (resp. decrement) the value of *w*_*c*_(*t*) (red curve in Fig 12). The activation of the *test* cell (blue events in Fig 12) results 4 time steps later in the spike of the Boolean cell ‘= 0’ or ‘≠ 0’ (red events in Fig 12), depending on whether *w*_*c*_(*t*) = 0 or *w*_*c*_(*t*) ≠ 0, respectively. During this process, the value of *w*_*c*_(*t*) is first incremented (2 time steps later) and then decremented (2 time steps later again) back to its original value. In other words, the testing procedure induces a back and forth fluctuation of *w*_*c*_(*t*), without finally modifying it from its initial value (this fluctuation is unfortunately unavoidable). A counter module is denoted as *counter*_*module*().

#### Detection modules

**Fig 13:**
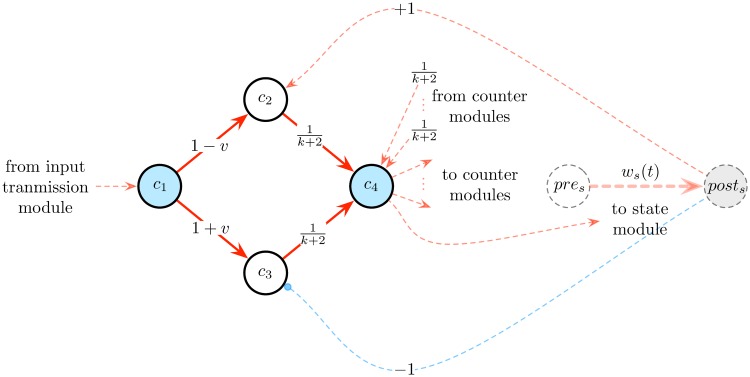
Detection module. This module is used to detect if the activation value of *post*_*s*_ is equal to *v* together with the fact that *k* signals from the counter modules are correctly received. If these conditions are fulfilled, the ‘detection cell’ *c*_4_ spikes, which triggers the simulation of the next transition of the machine. It is composed of 4 Boolean cells *c*_1_, *c*_2_, *c*_3_, *c*_4_ connected in a feedforward way.

*Detection modules* are used to retrieve—or detect—the current computational and counter states of the machine being simulated. This information is then employed to simulate the next transition of the machine. More precisely, each input symbol *a* ∈ Σ ∪ {*ϵ*}, computational state *q* ∈ *Q* and counter states ${\bar{c}}_{1},\ldots ,{\bar{c}}_{k}\in C$ of the machine are associated with a corresponding detection module. This module is activated if and only if the current input bit processed by the network is precisely *a*, the current synaptic weights *w*_*s*_(*t*) corresponds to the encoding of the computational state *q*, and the current synaptic weights $w_{c_{1}}\left(t\right),\ldots ,w_{c_{k}}\left(t\right)$ are the encodings of counter values with corresponding counter states ${\bar{c}}_{1},\ldots ,{\bar{c}}_{k}$. Afterwards, the detection module sends suitable activations to the state and counter modules so as to simulate the next transition $\delta \left(q,a,{\bar{c}}_{1},\ldots ,{\bar{c}}_{k}\right)=\left(q^{\prime },o_{1},\ldots ,o_{k}\right)$ of the machine. Formally, a detection module detects if the activation value of cell *post*_*s*_ of the state module is equal to a certain value *v*, together with the fact that *k* signals from cells = 0 or ≠ 0 of the *k* counter modules are correctly received. The module is composed of 4 Boolean cells connected in a feedforward manner, as illustrated in Fig 13. It is connected to the *input transmission module*, the *state module* and the *counter modules* described above.

**Fig 14:**
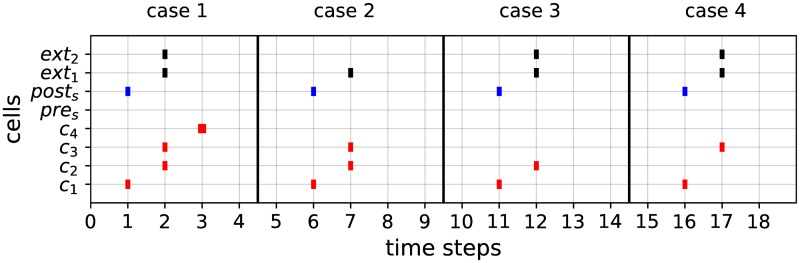
Examples of activity of the detection module. The detection module, composed of the cells *c*_1_, *c*_2_, *c*_3_, *c*_4_, receives activations from the state module via the cell *post*_*s*_, as well as from 2 counter modules via the cells *ext*_1_, *ext*_2_. The module detects whether the activation value of *post*_*s*_ is equal to *v* = 0.3 together with the fact that both *ext*_1_, *ext*_2_ have been activated. In case 1, these conditions are fulfilled, and thus the ‘detection cell’ *c*_4_ spikes (bold spike at *t* = 3). In all other cases, the required conditions are not fulfilled: either only one external activation is received (*ext*_2_ has not spiked, case 2), or the activation value *v* of *post*_*s*_ satisfies *v* > 0.3 (thus *c*_3_ is not spiking, case 3) or *v* < 0.3 (thus *c*_2_ is not spiking, case 4). In each case, the ‘detection cell’ *c*_4_ does not spike.

The activity of this module, illustrated in Fig 14, can be described as follows. Suppose that at time step *t*, cell *c*_1_ is spiking and cell *post*_*s*_ has an activation value of *v* (with 0 ≤ *v* ≤ 1). Then, at time *t* + 1, both *c*_2_ and *c*_3_ spike (since they receive signals of intensity 1). At next time *t* + 2, two signals of intensities $\frac{1}{k+2}$ are transmitted to *c*_4_. Suppose that at this same time step, *c*_4_ also receives *k* signals from the counter modules. Then, *c*_4_ receives *k* + 2 signals of intensities $\frac{1}{k+2}$, and hence spikes at time *t* + 3 (case 1 of Fig 14). By contrast, if at time step *t*, *c*_1_ is spiking and *post*_*s*_ has an activation value of *v*′ > *v* (resp. *v*′ < *v*), then at time *t* + 1 only *c*_2_ (resp. *c*_3_) spikes. Hence, at time *t* + 2, *c*_4_ receives less than *k* + 2 signals of intensities $\frac{1}{k+2}$, and thus stays quiet (cases 3 and 4 of Fig 14). Consequently, the ‘detection cell’ *c*_4_ (blue cell of Fig 13) spikes if and only if *post*_*s*_ has an exact activation value of *v* and *c*_4_ receives exactly *k* signals from its afferent connections. A detection module involving weights $1-v,1+v,\frac{1}{k+2}$ is denoted as *detection*_*module*(*v*, *k*).

#### Assembling the modules

Any given *k*-counter machine $C_{k}=\left(Q,\Sigma ,C,O,\delta ,0,F\right)$ (where Σ = {0, 1} and *Q* = {0, …, *n* − 1}) can be simulated by a recurrent neural network N subjected to the STDP rules given by Eqs (2) and (3). The network is obtained by a suitable assembling of the modules described above. The architecture of N is illustrated in Fig 5, and its detailed construction is given by Algorithm 1. In short, the network N is composed of 1 input encoding module (line 1), 1 input transmission module (line 2), 1 state module (line 3), *k* counter modules (lines 4–6) and at most |*Q*|⋅| Σ ∪ {*ϵ*}| ⋅ 2^*k*^ = 3*n*2^*k*^ detection modules (lines 7–11). The modules are connected together according to the patterns described in lines 12–47. This makes a total of $O(n2^{k})$ cells and $O(nk2^{k})$ synapses, which, since the number of counters *k* is fixed, corresponds to O(n) cells and O(n) synapses. A recurrent neural networks obtained via Algorithm 1 is referred to as an *STDP-based RNN*.

**Algorithm 1** Procedure which takes a *k*-counter machine as input and builds an STDP-based RNN that simulates it.

**Require**: *k*-counter machine $C_{k}=\left(Q,\Sigma ,C,O,\delta ,0,F\right)$, where Σ = {0, 1} and *Q* = {0, …, *n* − 1}

                     // note: computational states are represented as integers

                      // *** INSTANTIATION OF THE MODULES ***

1: IN1 ← *input*_*encoding*_*module*()                // input encoding module

2: IN2 ← *input*_*transmission*_*module*()             // input transmission module

3: ST ← *state*_*module*(*n* − 1)                // state module (where *n* = |*Q*|)

4: **for all** *j* = 1, …, *k* **do**

5:  C(*j*) ← *counter*_*module*()                   // *k* counter modules

6: **end for**

7: **for all** tuple $\left(i,a,{\bar{c}}_{1},\ldots ,{\bar{c}}_{k}\right)\in Q\times \Sigma \cup \left\{\epsilon \right\}\times C^{k}$ **do**

8:  **if** $\delta (i,a,{\bar{c}}_{1},\ldots ,{\bar{c}}_{k})$ is defined **then**

9:   $\text{DET}\left(i,a,{\bar{c}}_{1},\ldots ,{\bar{c}}_{k}\right)\leftarrow detection\_module\left(a_{min}+i\cdot \eta ,k\right)$    // detection modules

10:  **end if**

11: **end for**

                      // *** CONNECTION BETWEEN MODULES ***

12: connect *c*_19_ of IN1 to *u*_0_ of IN2: weight 1       // input encoding to input transmission

13: connect *c*_20_ of IN1 to *u*_1_ of IN2: weight 1

14: connect *tic* of IN1 to *u*_*ϵ*_ of IN2: weight 1

15: **for all** *j* = 1, …, *k* **do**                  // input transmission to counters

16:  connect *u*_0_, *u*_1_, *u*_*ϵ*_ of IN2 to *test* of C(*j*): weight 1

17: **end for**

18: connect *d*_2,0_, *d*_2,1_, *d*_2,*ϵ*_ of IN2 to *pre*_*s*_ of ST: weight 1       // input transmission to state

19: **for all** tuple $\left(i,a,{\bar{c}}_{1},\ldots ,{\bar{c}}_{k}\right)\in Q\times \Sigma \cup \left\{\epsilon \right\}\times C^{k}$ **do**

20:  **if** $\delta \left(i,a,{\bar{c}}_{1},\ldots ,{\bar{c}}_{k}\right)=\left(i^{\prime },o_{1},\ldots ,o_{k}\right)$ **then**

21:   connect *d*_3,*a*_ of IN2 to *c*_1_ of $\text{DET}(i,a,{\bar{c}}_{1},\ldots ,{\bar{c}}_{k})$: weight 1 // input transmission to detection

22:   connect *post* of ST to *c*_2_ of $\text{DET}(i,a,{\bar{c}}_{1},\ldots ,{\bar{c}}_{k})$: weight 1       // state to detection

23:   connect *post* of ST to *c*_3_ of $\text{DET}(i,a,{\bar{c}}_{1},\ldots ,{\bar{c}}_{k})$: weight −1

24:   **if** *a* ⩵ *ϵ* **then**                      //detection to input encoding

25:    connect *c*_4_ of $\text{DET}(i,a,{\bar{c}}_{1},\ldots ,{\bar{c}}_{k})$ to *c*_15_ of IN1: weight −1

26:   **end if**

27:   **if** *i*′ − *i* > 0 **then**                      // detection to state

28:    connect *c*_4_ of $\text{DET}(i,a,{\bar{c}}_{1},\ldots ,{\bar{c}}_{k})$ to *c*_3((*n*−1)−(*i*′−*i*))+1_ of ST: weight 1

29:   **else if** *i*′ − *i* < 0 **then**

30:    connect *c*_4_ of $\text{DET}(i,a,{\bar{c}}_{1},\ldots ,{\bar{c}}_{k})$ to ${\bar{c}}_{3(\left(n-1\right)-\left(i^{\prime }-i\right))+1}$ of ST: weight 1

31:   **end if**

32:   **for all** *j* = 1, …, *k* **do**                     // detection to counters

33:    **if** *o*_*j*_ ⩵ *push* **then**

34:     connect *c*_4_ of $\text{DET}(i,a,{\bar{c}}_{1},\ldots ,{\bar{c}}_{k})$ to cell *push* of C(j): weight 1

35:    **else if** *o*_*j*_ ⩵ *pop* **then**

36:     connect *c*_4_ of $\text{DET}(i,a,{\bar{c}}_{1},\ldots ,{\bar{c}}_{k})$ to cell *pop* of C(j): weight 1

37:    **end if**

38:   **end for**

39:  **end if**

40:  **for all** *j* = 1, …, *k* **do**                      // counters to detection

41:   **if** ${\bar{c}}_{j}==⊥$ **then**

42:    connect ‘= 0’ of C(*j*) to *c*_4_ of $\text{DET}(i,a,{\bar{c}}_{1},\ldots ,{\bar{c}}_{k})$: weight $\frac{1}{k+2}$

43:   **else if** ${\bar{c}}_{j}==⊤$ **then**

44:    connect ‘≠ 0’ of C(*j*) to *c*_4_ of $\text{DET}(i,a,{\bar{c}}_{1},\ldots ,{\bar{c}}_{k})$: weight $\frac{1}{k+2}$

45:   **end if**

46:  **end for**

47: **end for**

### Turing completeness

We now prove that any *k*-counter machine is correctly simulated by its corresponding STDP-based RNN given by Algorithm 1. Since 2-counter machines are Turing complete, then so is the class of STDP-based RNNs. Towards this purpose, the following definitions need to be introduced.

Let N be an STDP-based RNN. The input cells of N are the cells *in*_0_, *in*_1_, *end*, *tic* of the input encoding module (cf. Fig 6, four blue cells of the first layer). Thus, inputs of N are vectors in $B^{4}$ whose successive components represent the spiking configurations of cells *in*_0_, *in*_1_, *end*, and *tic*, respectively. In order to describe the input streams of N, we consider the following vectors of $B^{4}$:

$$0≔\left(\begin{array}{c} 1\\ 0\\ 0\\ 0 \end{array}\right),1≔\left(\begin{array}{c} 0\\ 1\\ 0\\ 0 \end{array}\right),end≔\left(\begin{array}{c} 0\\ 0\\ 1\\ 0 \end{array}\right),tic_{i}≔\left(\begin{array}{c} 0\\ 0\\ 0\\ 1 \end{array}\right),\text{for}\;\text{all}\;i\geq 0\;\text{and}\;\emptyset ≔\left(\begin{array}{c} 0\\ 0\\ 0\\ 0 \end{array}\right)$$

According to these notations, the input stream **0011end∅∅tic** corresponds to the following sequence of vectors provided at successive time steps

$$\left(\begin{array}{c} 1\\ 0\\ 0\\ 0 \end{array}\right)\left(\begin{array}{c} 1\\ 0\\ 0\\ 0 \end{array}\right)\left(\begin{array}{c} 0\\ 1\\ 0\\ 0 \end{array}\right)\left(\begin{array}{c} 0\\ 1\\ 0\\ 0 \end{array}\right)\left(\begin{array}{c} 0\\ 0\\ 1\\ 0 \end{array}\right)\left(\begin{array}{c} 0\\ 0\\ 0\\ 0 \end{array}\right)\left(\begin{array}{c} 0\\ 0\\ 0\\ 0 \end{array}\right)\left(\begin{array}{c} 0\\ 0\\ 0\\ 1 \end{array}\right)$$

i.e., to the successive spikes of cells *in*_0_, *in*_0_, *in*_1_, *in*_1_, *end*, followed by two times steps during which all cells are quiet, followed by a last spike of the cell *tic*.

**Fig 15:**
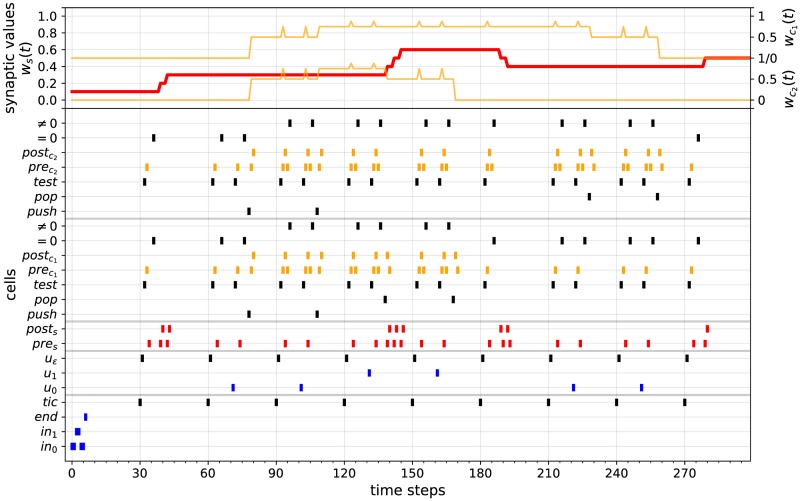
Simulation 1. Computation of the STDP-based RNN simulating the 2-counter machine of Fig 4 over input 001100. The lower graph is a raster plot displaying the spiking patterns of some of the cells of the network belonging to the input encoding module (cells *in*_0_, *in*_1_, *end*, *tic*), the input transmission module (cells *u*_0_, *u*_1_, *u*_*ϵ*_), the state module (cells *pres*_*s*_, *post*_*s*_) and the two counter modules (cells *push*, *pop*, *test*, $pre_{c_{k}}$, $post_{c_{k}}$, = 0, ≠ 0, for *k* = 1,2). The upper graph displays the evolution of the synaptic weights *w*_*s*_(*t*) (red curve) and $w_{c_{1}}(t),w_{c_{2}}(t)$ (orange curves) over time. The red curve is displayed relatively to the left-hand scale (ranging from 0 to 1). The two orange curves are are displayed relatively to the upper and lower right-hand scales, respectively (both ranging from 0 to 1). The evolution of *w*_*s*_(*t*) and $w_{c_{1}}(t),w_{c_{2}}(t)$ (red and orange curves) represent the encodings of the successive states and counter values of the 2-counter machine, respectively.

For any binary input *w* = *a*_0_ ⋯ *a*_*p*_ ∈ Σ*, let $u_{w}\in {\left(B^{4}\right)}^{*}$ be the corresponding input stream of N defined by

$$\begin{array}{c} u_{w}=a_{0}\cdots a_{p}\text{end}\underset{K_{p+1}^{\prime }}{\underset{︸}{\emptyset \cdots \emptyset }}{\text{tic}}_{0}\underset{K}{\underset{︸}{\emptyset \cdots \emptyset }}{\text{tic}}_{1}\underset{K}{\underset{︸}{\emptyset \cdots \emptyset }}{\text{tic}}_{2}\cdots \end{array}$$

where **a**_**i**_ = **0** if *a*_*i*_ = 0 and **a**_**i**_ = **1** if *a*_1_ = 0, for *i* = 0, …, *p*. In other words, the input stream *u*_*w*_ consists of successive spike from cells *in*_0_ and *in*_1_ (inputs **a**_**0**_ ⋯ **a**_**p**_), followed by one spike from cell *end* (input **end**), followed by $K_{p+1}^{\prime }$ time steps during which nothing happens (inputs **∅**⋯**∅**), followed by successive spikes from cell *tic*, interspersed by constant intervals of *K* time steps during which nothing happens (input blocks **tic**_**i**_**∅** ⋯ **∅**). The value of $K_{p+1}^{\prime }$ is chosen such that, at time step *p* + 2 + *K*′, the *p* + 1 successive bits of *u*_*w*_ are correctly stored into cell *c*_14_ of the input encoding module. The value of *K* is chosen such that, after each spike of the *tic* cell, the updating of the state and counter modules can be achieved within *K* time steps. Taking $K_{p+1}^{\prime }\geq 3\left(p+1\right)+4$ and *K* ≥ 17 + 3(*n* − 1) (where *n* = |*Q*|) satisfies these requirements. Note that $K_{p+1}^{\prime }$ depends on the input length, while *K* is constant (for a given counter machine). An input stream of this form is depicted by the 4 bottom lines of Fig 15 (in this case $K_{p+1}^{\prime }=23$ and *K* = 29). Besides, for each *i* ≥ 0, let *t*_*i*_ be the time step at which **tic**_**i**_ occurs. For instance, in Fig 15, one has *t*_0_ = 30, *t*_1_ = 60, *t*_2_ = 90, *t*_3_ = 120, …. Let also

$$\begin{array}{ccc} & & w_{s,i}≔w_{s}\left(t_{i}-1\right)\\ & & w_{c_{1,i}}≔w_{c_{1}}\left(t_{i}-1\right),\ldots ,w_{c_{k,i}}≔w_{c_{k}}\left(t_{i}-1\right) \end{array}$$

be the synaptic weights $w_{s}\left(t\right),w_{c_{1}}\left(t\right),\ldots ,w_{c_{k}}\left(t\right)$ of the state and counter modules at time step *t*_*i*_ − 1 (i.e., 1 time step before **tic**_**i**_ has occurred), with the assumption that

$$\begin{array}{c} \left(w_{s,0},w_{c_{1},0},\ldots ,w_{c_{k},0}\right)=\left(a_{min},0,\ldots ,0\right). \end{array}$$

For example, in Fig 15, the values of $w_{s}\left(t\right),w_{c_{1}}\left(t\right),w_{c_{2}}\left(t\right)$ over time are represented by the upper red and orange curves (pay attention to the different left-hand and right-hand scales associated to these curves): one has $\left(w_{s,0},w_{c_{1},0},w_{c_{2},0}\right)=\left(0.1,0,0\right),\;\left(w_{s,1},w_{c_{1},1},w_{c_{2},1}\right)=\left(0.3,0,0\right),\;\left(w_{s,2},w_{c_{1},2},w_{c_{2},2}\right)=\left(0.3,0.5,0.5\right),\;\left(w_{s,3},w_{c_{1},3},w_{c_{2},3}\right)=\left(0.3,0.75,0.75\right)$, etc. Furthermore, let $a_{i}^{\prime \prime }\in \Sigma \cup \left\{\epsilon \right\}\cup \left\{\emptyset \right\}$ be defined by

$$\begin{array}{c} a_{i}^{\prime \prime }=\{\begin{array}{cc} a & \text{if}\;\text{cell}\;c_{4}\;\text{of}\;\text{one}\;\text{and}\;\text{only}\;\text{one}\;\text{detection}\;\text{module}\;\text{DET}(q,a,{\bar{c}}_{1},\ldots ,{\bar{c}}_{k})\\ & \text{spikes}\;\text{between}\;t_{i}\;\text{and}\;t_{i+1}\text{,}\;\text{for}\;\text{some}\;q\in Q\text{,}\;a\in \Sigma \cup \{\epsilon \}\text{,}\;{\bar{c}}_{1},\ldots ,{\bar{c}}_{k}\in C\\ \emptyset & \text{otherwise} \end{array} \end{array}$$

In other words, $a_{i}^{\prime \prime }$ is the *input symbol* (possibly *ϵ*) processed by N between *t*_*i*_ and *t*_*i*+1_. For instance, in Fig 15, the successive input bits processed by the network are displayed by the spiking patterns of the cells *u*_*ϵ*_, *u*_0_, *u*_1_: one has $a_{0}^{\prime \prime }=\epsilon$ (only *u*_*ϵ*_ spikes between *t*_0_ and *t*_1_), $a_{1}^{\prime \prime }=0$ (both *u*_*ϵ*_ and then *u*_0_ spike between *t*_1_ and *t*_2_, but only *u*_0_ leads to the activation of a detection module, even if this is not represented), $a_{2}^{\prime \prime }=0$ (*u*_0_ spikes after *u*_*ϵ*_ between *t*_2_ and *t*_3_), $a_{3}^{\prime \prime }=1$ (*u*_1_ spikes after *u*_*ϵ*_ between *t*_3_ and *t*_4_), etc.

Now, for any input stream *u*_*w*_, the *computation* of N over *u*_*w*_ is the sequence

$$\begin{array}{c} N\left(u_{w}\right)={\left((w_{s,i},a_{i}^{\prime \prime },w_{c_{1,i}},\ldots ,w_{c_{k,i}})\right)}_{i=0}^{l_{2}},\;\;l_{2}\in N\cup \left\{\infty \right\} \end{array}$$ (5)

where $l_{2}=\text{min}\left\{t_{i}:a_{i}^{\prime \prime }=\emptyset ,i\geq 0\right\}$. In other words, the computation of N over *u*_*w*_ is the sequence of successive values of $w_{s}\left(t\right),a_{i}^{\prime \prime },w_{c_{1}}\left(t\right),\ldots ,w_{c_{k}}\left(t\right)$, which are supposed to encode the successive states, input symbols and counter values of the machine to be simulated, respectively.

According to these considerations, we say that $C_{k}$ *is simulated in real time by* N, or equivalently that N *simulates* $C_{k}$ *in real time*, if and only if, for any input *w* ∈ Σ* with corresponding input stream $u_{w}\in {\left(B^{4}\right)}^{*}$, the computations of $C_{k}$ over *w* (Eq (4)) and of N over *u*_*w*_ (Eq (5))

$$\begin{array}{ccc} C_{k}\left(w\right) & = & {\left((n_{i},a_{i}^{\prime },c_{1,i},\ldots ,c_{k,i})\right)}_{i=0}^{l_{1}}\\ N(u_{w}) & = & {\left((w_{s,i},a_{i}^{\prime \prime },w_{c_{1,i}},\ldots ,w_{c_{k,i}})\right)}_{i=0}^{l_{2}} \end{array}$$

satisfy the following conditions:

$$w_{s,i}=a_{min}+n_{i}\cdot \eta \;\text{state}\;\text{condition}$$ (6)

$$a_{i}^{\prime \prime }=a_{i}^{\prime }\;\text{symbol}\;\text{condition}$$ (7)

$$w_{c_{j,i}}=r_{c_{j,i}}\;\text{for}\;\text{all}\;j=1,\ldots ,k\;\text{counter}\;\text{values}\;\text{condition}$$ (8)

for all *i* = 0, …, *l*_1_, which implicitly implies that *l*_2_ ≥ *l*_1_ (recall that *r*_0_ ≔ 0 and $r_{n}≔\sum_{i=1}^{n}\frac{1}{2^{i}}$, for all *n* > 0). In other words, $C_{k}$ is simulated by N iff, on every input, the computations of $C_{k}$ is perfectly reflected by that of N: the sequence of input symbols processed by $C_{k}$ and N coincide (Condition (7)), and the successive computational states and counter values of $C_{k}$ are properly encoded into the successive synaptic weights of $w_{s}\left(t\right),w_{c_{1}}\left(t\right),\ldots ,w_{c_{k}}\left(t\right)$ of N, respectively (Conditions (6) and (8)). According to these considerations, each state $n_{i}\in N$ and counter value $c_{j,i}\in N$ of $C_{k}$ is encoded by the synaptic value $w_{s}\left(t_{i}-1\right)=a_{min}+n_{i}\cdot \eta \in Q$ and $w_{c_{j}}\left(t_{i}-1\right)=r_{c_{j,i}}\in Q$, for *j* = 1, …, *k*, respectively. The real time aspect of the simulation is ensured by the fact that the successive time steps (*t*_*i*_)_*i*≥0_ involved in the computation N(w) are separated by a constant number of time steps *K* > 0. This means that the transitions of $C_{k}$ are simulated by N in fixed amount of time.

We now show that, in this precise sense, any *k*-counter machine is correctly simulated its corresponding STDP-based recurrent neural network.

**Theorem 1**. *Let* $C_{k}$ *be a k*-*counter machine and* N *be the STDP*-*based RNN given by Algorithm 1 applied on* $C_{k}$. *Then*, $C_{k}$ *is simulated in real time by* N.

*Proof*. Let *w* = *a*_0_ ⋯ *a*_*p*_ ∈ Σ* be some input and $u_{w}\in {\left(B^{4}\right)}^{*}$ be its corresponding input stream. Consider the two computations of $C_{k}$ on *w* (Eq (4)) and of N on *u*_*w*_ (Eq (5)), respectively:

$$\begin{array}{ccc} C_{k}\left(w\right) & = & {\left((n_{i},a_{i}^{\prime },c_{1,i},\ldots ,c_{k,i})\right)}_{i=0}^{l_{1}}\\ N(u_{w}) & = & {\left((w_{s,i},a_{i}^{\prime \prime },w_{c_{1,i}},\ldots ,w_{c_{k,i}})\right)}_{i=0}^{l_{2}}. \end{array}$$

We prove by induction on *i* that $C_{k}\left(w\right)$ and $N(u_{w})$ satisfy Conditions (6)–(8), for all *i* = 0, …, *l*_1_.

By definition, the first elements of $C_{k}\left(w\right)$ and $N(u_{w})$ are

$$\begin{array}{ccc} \left(n_{0},a_{0}^{\prime },c_{1,0},\ldots ,c_{k,0}\right) & = & \left(0,a_{0}^{\prime },0,\ldots ,0\right)\\ \left(w_{s,0},a_{0}^{\prime \prime },w_{c_{1},0},\ldots ,w_{c_{k},0}\right) & = & (a_{min},a_{0}^{\prime \prime },0,\ldots ,0). \end{array}$$

Hence, Conditions (6) and (8) are satisfied for *i* = 0, i.e.,

$$\begin{array}{c} w_{s,0}=a_{min}+n_{0}\cdot \eta \;\;\text{and}\;\;w_{c_{j,0}}=r_{c_{j,0}}\;\text{for}\;\text{all}\;j=1,\cdots ,k. \end{array}$$ (9)

We now prove Condition (7) for *i* = 0. Towards this purpose, the following observations are needed. By construction and according to the value of *K*_*p*+1_, at time *t*_0_ − 1, cell *c*_14_ of the input encoding module IN1 holds the encoding of the whole input *w* = *a*_0_ ⋯ *a*_*p*_ (the latter being considered as a stack). The top element of this stack is *a*_0_. Besides, according to Relations (9) and Algorithm 1 (lines 22–23 and 40–46), only the detection modules $\text{DET}(n_{0},a,{\bar{c}}_{1,0},\ldots ,{\bar{c}}_{k,0})$, where *a* ∈ Σ ∪ {*ϵ*}, are susceptible have their cell *c*_4_ activated between *t*_0_ and *t*_1_ (indeed, only these modules are capable of “detecting” the current synaptic value *a*_*min*_ + *n*_0_ ⋅ *η* and counters states $c_{1,0},\ldots ,c_{k,0}$ involved in Relations (9)).

Now, consider the symbol $a_{0}^{\prime }\in \Sigma \cup \left\{\epsilon \right\}$. Then either $a_{0}^{\prime }\in \Sigma$ or $a_{0}^{\prime }=\epsilon$. As a first case, suppose that $a_{0}^{\prime }\in \Sigma$. Since $a_{0}^{\prime }\neq \epsilon$ and $a_{0}^{\prime }$ is the first symbol processed by $C_{k}$ during its computation over input *w* = *a*_0_ ⋯ *a*_*p*_ (cf. Eq (4)), one necessarily has $a_{0}^{\prime }=a_{0}$. Thus, $\delta \left(n_{0},a_{0}^{\prime },{\bar{c}}_{1,0},\ldots ,{\bar{c}}_{k,0}\right)=\delta \left(n_{0},a_{0},{\bar{c}}_{1,0},\ldots ,{\bar{c}}_{k,0}\right)$, and the determinism of $C_{k}$ ensures that $\delta (n_{0},\epsilon ,{\bar{c}}_{1,0},\ldots ,{\bar{c}}_{k,0})$ is undefined. According to Algorithm 1 (lines 7–11), the module $\text{DET}(n_{0},a_{0},{\bar{c}}_{1,0},\ldots ,{\bar{c}}_{k,0})$ is instanciated, whereas $\text{DET}(n_{0},\epsilon ,{\bar{c}}_{1,0},\ldots ,{\bar{c}}_{k,0})$ is not. Hence, the dynamics of N between *t*_0_ and *t*_1_ goes as follows. At time *t*_0_, the cell *tic* of IN1 sends a signal to *u*_*ϵ*_ of IN2 (Algorithm 1, line 14) which propagates to the detection modules associated to symbol *ϵ* (Algorithm 1, line 21). Since the module $\text{DET}(n_{0},\epsilon ,{\bar{c}}_{1,0},\ldots ,{\bar{c}}_{k,0})$ does not exist, it can certainly not be activated, and thus, the cell *c*_15_ of IN1 will not be inhibited in return (Algorithm 1, line 24–26). The spike of *c*_15_ will then trigger the sub-circuit of IN1 that pops the top element of the stack currently encoded in *c*_14_, namely, the symbol *a*_0_. This triggers the activation of *c*_19_ or *c*_20_ of IN1 depending on whether *a*_0_ = 0 or *a*_0_ = 1. This activity then propagates to cells $u_{a_{0}}$ and next $d_{3,a_{0}}$ of IN2 (Algorithm 1, lines 12–13). It propagates further to the detection modules of the form DET(⋅, *a*_0_, ⋅, …, ⋅), and in particular to $\text{DET}(n_{0},a_{0},{\bar{c}}_{1,0},\ldots ,{\bar{c}}_{k,0})$ (Algorithm 1, line 21). According to Relations (9), the cell *c*_4_ of $\text{DET}(n_{0},a_{0},{\bar{c}}_{1,0},\ldots ,{\bar{c}}_{k,0})$, and of this module only, will be activated, since it is the only module of this form capable of detecting the current weight *w*_*s*_(*t*) = *a*_*min*_ + *η* ⋅ *n*_0_ as well as the current counter states ${\bar{c}}_{1,0},\ldots ,{\bar{c}}_{k,0}$ (Algorithm 1, lines 22–23 and 40–46). This amounts to saying that the symbol $a_{0}^{\prime \prime }$ processed by N between *t*_0_ and *t*_1_ is equal to *a*_0_. Therefore, $a_{0}^{\prime \prime }=a_{0}=a_{0}^{\prime }$. This shows that in this case, Condition (7) holds for *i* = 0.

As a second case, suppose that $a_{0}^{\prime }=\epsilon$. It follows that $\delta \left(n_{0},a_{0}^{\prime },{\bar{c}}_{1,0},\ldots ,{\bar{c}}_{k,0}\right)=\delta \left(n_{0},\epsilon ,{\bar{c}}_{1,0},\ldots ,{\bar{c}}_{k,0}\right)$, and by Algorithm 1 (lines 7–11), the module $\text{DET}(n_{0},\epsilon ,{\bar{c}}_{1,0},\ldots ,{\bar{c}}_{k,0})$ is instanciated. Consequently, the dynamics of N between *t*_0_ and *t*_1_ goes as follows. At time *t*_0_, the cell *tic* of IN1 sends a signal to *u*_*ϵ*_ of IN2 (Algorithm 1, line 14) which propagates to the module $\text{DET}(n_{0},\epsilon ,{\bar{c}}_{1,0},\ldots ,{\bar{c}}_{k,0})$ (Algorithm 1, line 21). By Relations (9), the cell *c*_4_ of this detection module, and of only this one, will be activated (Algorithm 1, lines 22–23 and 40–46). This amounts to saying that $a_{0}^{\prime \prime }=\epsilon =a_{0}^{\prime }$. Therefore, in this case also, Condition (7) holds for *i* = 0.

For the induction step, let *m* < *l*_1_, and suppose that Conditions (6)–(8) are satisfied for all *i* ≤ *m*. Let also *o*_1,*m*+1_, …, *o*_*k*,*m*+1_ ∈ *O* be the counter operations such that

$$\begin{array}{c} \delta \left(n_{m},a_{m}^{\prime },{\bar{c}}_{1,m},\ldots ,{\bar{c}}_{k,m}\right)=\left(n_{m+1},o_{1,m+1},\ldots ,o_{k,m+1}\right). \end{array}$$ (10)

By definition of the sequence $C_{k}\left(w\right)$, $a_{m}^{\prime }\in \Sigma \cup \left\{\epsilon \right\}$ and the counter operations satisfy

$$\begin{array}{c} c_{1,m+1}=o_{1,m+1}\left(c_{1,m}\right),\ldots ,c_{k,m+1}=o_{k,m+1}\left(c_{k,m}\right). \end{array}$$ (11)

By the induction hypothesis (Condition (7)), $a_{m}^{\prime \prime }=a_{m}^{\prime }$. The definition of $a_{m}^{\prime \prime }$ ensures that the cell *c*_4_ of one and only one detection module $\text{DET}(q,a_{m}^{\prime \prime },{\bar{c}}_{1},\ldots ,{\bar{c}}_{k})$ is activated between time steps *t*_*m*_ and *t*_*m*+1_, for some *q* ∈ *Q* and some ${\bar{c}}_{1},\ldots ,{\bar{c}}_{k}\in C$. But by the induction hypotheses (Conditions (6) and (8)), at time step *t*_*m*_ − 1, one has

$$\begin{array}{ccc} w_{s,m} & = & a_{min}+n_{m}\cdot \eta \end{array}$$ (12)

$$\begin{array}{ccc} w_{c_{j},m} & = & r_{c_{j,m}},\;\text{for}\;\text{all}\;j=1,\ldots ,k. \end{array}$$ (13)

Hence, Relations (12) and (13) and Algorithm 1 (lines 22–23 and 40–46) ensure that the module $\text{DET}(n_{m},a_{m}^{\prime \prime },{\bar{c}}_{1,m},\ldots ,{\bar{c}}_{k,m})$, and only this one, has its cell *c*_4_ activated between time steps *t*_*m*_ and *t*_*m*+1_. By Relation (10), the cell *c*_4_ of this detection module is connected to cell

$$\begin{array}{ccc} c_{3(\left(n-1\right)-\left(n_{m+1}-n_{m}\right))+1} & \;\text{if}\; & n_{m+1}-n_{m}>0\;\;\text{or}\;\\ {\bar{c}}_{3(\left(n-1\right)-\left(n_{m+1}-n_{m}\right))+1} & \;\text{if}\; & n_{m+1}-n_{m}<0 \end{array}$$

of the state module ST (Algorithm 1, lines 27–31). Hence, the activation of this detection module between *t*_*m*_ and *t*_*m*+1_ induces subsequent spiking patterns of the state module which, by construction, increments (if *n*_*m*+1_ − *n*_*m*_ > 0) or decrements (if *n*_*m*+1_ − *n*_*m*_ < 0) the synaptic weight *w*_*s*_(*t*) by |*n*_*m*+1_ − *n*_*m*_| ⋅ *η*, and hence, changes it from its current value *a*_*min*_ + *n*_*m*_ ⋅ *η* (cf. Eq (12)) to the new value *a*_*min*_ + *n*_*m*_ ⋅ *η* + (*n*_*m*+1_ − *n*_*m*_) ⋅ *η* = *a*_*min*_ + *n*_*m*+1_ ⋅ *η*. Note that each spiking pattern takes 3 time steps, and hence, the updating of *w*_*s*_(*t*) takes at most 3(*n* − 1) time steps, where *n* is the number of states of $C_{k}$ (the longest update being when |*n*_*m*+1_ − *n*_*m*_| = *n* − 1, which takes 3(*n* − 1) time steps). Therefore, at time *t*_*m*+1_ − 1, one has

$$\begin{array}{c} w_{s,m+1}=a_{min}+n_{m+1}\cdot \eta . \end{array}$$

This shows that Condition (6) is satisfied for *i* = *m* + 1.

Similarly, by Relation (10), the cell *c*_4_ of the module $\text{DET}(n_{m},a_{m}^{\prime \prime },{\bar{c}}_{1,m},\ldots ,{\bar{c}}_{k,m})$ is connected to cells *push* or *pop* of the counter module C(*j*) depending on whether *o*_*j*,*m*+1_ ⩵ *push* or *o*_*j*,*m*+1_ ⩵ *pop*, respectively, for *j* = 1, …, *k* (Algorithm 1, lines 32–38). Hence, the activation of the detection module $\text{DET}(n_{m},a_{m}^{\prime \prime },{\bar{c}}_{1,m},\ldots ,{\bar{c}}_{k,m})$ between *t*_*m*_ and *t*_*m*+1_ induces subsequent activations of the counter modules which, by construction, change the synaptic weights $w_{c_{j}}\left(t\right)$ from their current value $r_{c_{j,m}}$ to $r_{o_{j,m+1}(c_{j,m})}$, for *j* = 1, …, *k*. Note that the updating of each $w_{c_{j}}\left(t\right)$ takes only 3 time steps. Consequently, at time *t*_*m*+1_ − 1, one has

$$\begin{array}{c} w_{c_{j,m+1}}=r_{o_{j,m+1}\left(c_{j,m}\right)},\;\text{for}\;j=1,\cdots ,k \end{array}$$

By Relation (11), these equations can be rewritten as

$$\begin{array}{c} w_{c_{j,m+1}}=r_{c_{j,m+1}},\;\text{for}\;j=1,\cdots ,k. \end{array}$$

This shows that Condition (8) is satisfied for *i* = *m* + 1.

We now show that Condition (7) holds for *i* = *m* + 1. By the induction hypothesis, one has ${\left(a_{i}^{\prime }\right)}_{i=0}^{m}={\left(a_{i}^{\prime \prime }\right)}_{i=0}^{m}$. We must prove that $a_{m+1}^{\prime }=a_{m+1}^{\prime \prime }$. By definition, elements from ${\left(a_{i}^{\prime }\right)}_{i=0}^{m}$ and ${\left(a_{i}^{\prime \prime }\right)}_{i=0}^{m}$ belong to Σ ∪ {*ϵ*}. Let ${\left(a_{i_{j}}^{\prime }\right)}_{j=0}^{p_{1}}$ (with *p*_1_ ≤ *m*) and ${\left(a_{i_{j}}^{\prime \prime }\right)}_{j=0}^{p_{2}}$ (with *p*_2_ ≤ *m*) be the subsequences formed by the non-empty symbols of ${\left(a_{i}^{\prime }\right)}_{i=0}^{m}$ and ${\left(a_{i}^{\prime \prime }\right)}_{i=0}^{m}$, respectively. The induction hypothesis ensures that *p*_1_ = *p*_2_ = *p*′ and

$$\begin{array}{c} {\left(a_{i_{j}}^{\prime }\right)}_{j=0}^{p^{\prime }}={\left(a_{i_{j}}^{\prime \prime }\right)}_{j=0}^{p^{\prime }}. \end{array}$$ (14)

Moreover, by definition again, ${\left(a_{i}^{\prime }\right)}_{i=0}^{m}$ is the sequence of empty and non-empty symbols processed by $C_{k}$ during the *m* + 1 first steps of its computation over input *w* = *a*_0_⋯*a*_*p*_ (cf. Eq (4)). Hence, the subsequence of its non-empty symbols ${\left(a_{i_{j}}^{\prime }\right)}_{j=0}^{p^{\prime }}$ corresponds precisely to the *p*′ + 1 successive letters of *w*, i.e.,

$$\begin{array}{c} {\left(a_{i_{j}}^{\prime }\right)}_{j=0}^{p^{\prime }}={\left(a_{i}\right)}_{i=0}^{p^{\prime }}. \end{array}$$ (15)

The fact that *ϵ* symbols of ${\left(a_{i}^{\prime }\right)}_{i=0}^{m}$ vanish within concatenation together with Relation (15) yield the following equalities

$$\begin{array}{c} a_{0}^{\prime }\cdots a_{m}^{\prime }=a_{i_{0}}^{\prime }\cdots a_{i_{p^{\prime }}}^{\prime }=a_{0}\cdots a_{p^{\prime }}. \end{array}$$ (16)

Also, Relations (14) and (15) directly imply

$$\begin{array}{c} {\left(a_{i_{j}}^{\prime \prime }\right)}_{j=0}^{p^{\prime }}={\left(a_{i}\right)}_{i=0}^{p^{\prime }}. \end{array}$$ (17)

Besides, as already mentioned, at time *t*_0_ − 1, cell *c*_14_ of module IN1 holds the encoding of input *w* = *a*_0_⋯*a*_*p*_ (considered as a stack). Between times *t*_0_ and *t*_*m*+1_ − 1, the elements of ${\left(a_{i}^{\prime \prime }\right)}_{i=0}^{m}$ are successively processed by N (cf. Eq (5)). During this time interval, the successive non-empty symbols of ${\left(a_{i}^{\prime \prime }\right)}_{i=0}^{m}$, i.e., the elements of ${\left(a_{i_{j}}^{\prime \prime }\right)}_{j=0}^{p^{\prime }}={\left(a_{i}\right)}_{i=0}^{p^{\prime }}$ (cf. Relation (17)), are successively popped from *w* = *a*_0_⋯*a*_*p*_ and the remaining string stored in cell *c*_14_. Consequently, at time *t*_*m*+1_ − 1, cell *c*_14_ holds the encoding of the remaining string *a*_*p*′+1_⋯*a*_*p*_, and thus, its top element is *a*_*p*′+1_.

From this point onwards, the proof of Relation (7) for the case *i* = 0 can be adapted to the present situation. In short, consider $a_{m+1}^{\prime }\in \Sigma \cup \left\{\epsilon \right\}$. Then either $a_{m+1}^{\prime }\in \Sigma$ or $a_{m+1}^{\prime }=\epsilon$. Note that in case $a_{m+1}^{\prime }\in \Sigma$, Relation (16) ensures that $a_{m+1}^{\prime }=a_{p^{\prime }+1}$. Taking this fact into account and replacing variables $a_{0},a_{0}^{\prime },a_{0}^{\prime \prime },n_{0},{\bar{c}}_{1,0},\ldots ,{\bar{c}}_{k,0}$ of the previous argument by $a_{m+1},a_{m+1}^{\prime },a_{m+1}^{\prime \prime },n_{m+1},{\bar{c}}_{1,m+1},\ldots ,{\bar{c}}_{k,m+1}$, respectively, leads to $a_{m+1}^{\prime \prime }=a_{m+1}^{\prime }$. Therefore, Condition (7) holds for *i* = *m* + 1.

Finally, we show that STDP-based RNNs are Turing complete. Let N be an STDP-based RNN. Let also acc,rej∈Q be two specific values for *w*_*s*_(*t*). For any binary input *w* = *a*_0_⋯*a*_*p*_ ∈ Σ*, we say that *w* is *accepted* (resp. *rejected*) by N if the sequence $N(u_{w})$ is finite, and its last element $\left(w_{s,l_{2}},a_{l_{2}}^{\prime \prime },w_{c_{1,l_{2}}},\ldots ,w_{c_{k,l_{2}}}\right)$ satisfies $a_{l_{2}}^{\prime \prime }=\epsilon$ and $w_{s,l_{2}}=acc$ (resp. $w_{s,l_{2}}=rej$). The *language recognized by N*, denoted by L(N), is the set of inputs accepted by N. A language *L* ⊆ Σ* is *recognizable* by some STDP-based RNN if there exists some STDP-based RNN N such that L(N)=L.

**Corollary 1**. *Let L* ⊆ Σ* *be some language*. *The language L is recognizable by some Turing machine if and only if L is recognizable by some STDP-based RNN*.

*Proof*. Suppose that *L* is recognizable by some STDP-based RNN N. The construction described in Algorithm 1 ensures that N can be simulated by some Turing machine M. Hence, *L* is recognizable by some Turing machine M. Conversely, suppose that *L* is recognizable by some Turing machine M. Then *L* is also recognizable by some 2-counter machine $C_{2}$ [57]. By Theorem 1, *L* is recognizable by some STDP-based RNN N.

### Simulations

We now illustrate the correctness of our construction by means of computer simulations.

**Fig 16:**
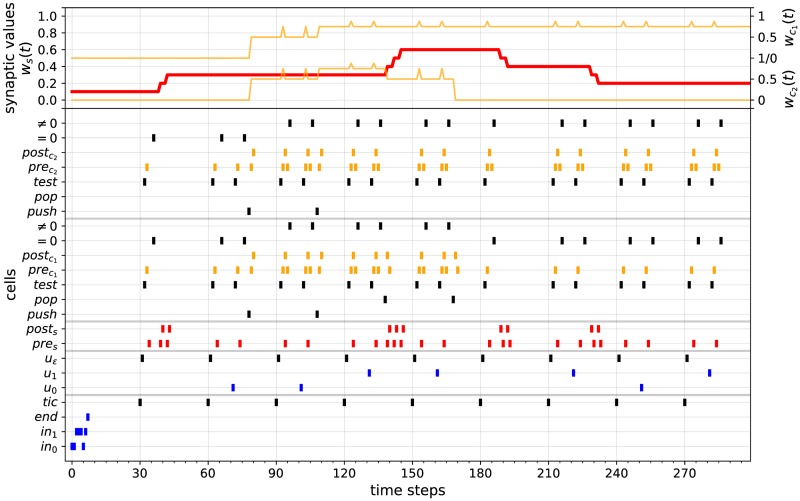
Simulation 2. Computation of the STDP-based RNN simulating the 2-counter machine of Fig 4 over input 0011101.

First, let us recall that the 2-counter machine of Fig 4 recognizes the recursively enumerable (but non context-free and non regular) language {0^*n*^1^*n*^0^*n*^: *n* > 0}, i.e., the sequences of bits beginning with a strictly positive number of 0’s followed by the same number of 1’s and followed again by the same number of 0’s. For instance, inputs *w*_1_ = 001100 and *w*_2_ = 0011101 are respectively accepted and rejected by the machine. Based on the previous considerations, we implemented an STDP-based RNN simulating this 2-counter machine. The network contains 390 cells connected together according to the construction given by Algorithm 1. We also set *a*_*min*_ = *η* = 0.1 in the STDP rule of Eq (2). Two computations of this network over an accepting and a rejecting input stream are illustrated in Figs 15 and 16. These simulations illustrate the correctness of the construction described in Algorithm 1.

More specifically, the computation of the network over the input stream

$$\begin{array}{c} u_{w_{1}}=001100\;\text{end}\underset{K_{6}^{\prime }=23}{\underset{︸}{\emptyset \cdots \emptyset }}{\text{tic}}_{0}\underset{K=29}{\underset{︸}{\emptyset \cdots \emptyset }}{\text{tic}}_{1}\underset{K=29}{\underset{︸}{\emptyset \cdots \emptyset }}{\text{tic}}_{2}\cdots \end{array}$$

which corresponds to the encoding of *w*_1_ = 001100, is displayed in Fig 15. In this case, taking *K* = 17 + 3(5 − 1) = 29 suffices for the correctness of the simulation (since the largest possible state update, in terms of the states’ indices, is a change from *q*_5_ to *q*_1_). The lower raster plot displays the spiking activities of some of the cells of the network belonging to the input encoding module (*in*_0_, *in*_1_, *end*, *tic*), the input transmission module (*u*_0_, *u*_1_, *u*_*ϵ*_), the state module (*pres*_*s*_, *post*_*s*_) and the two counter modules ($push,pop,test,pre_{c_{k}},post_{c_{k}},=0,\neq 0$, for *k* = 1, 2).

From time step *t* = 0 to *t* = 6, the encoding of the input stream 001100 is transmitted to the network via activations of cells *in*_0_, *in*_1_ and *end* (blue pattern). Between *t* = 6 and *t* = 30, the input pattern is encoded into activation values of sigmoid cells in the input encoding module, as illustrated in Fig 7. From *t* = 30 onwards, the *tic* cell is activated every 30 time steps in order to trigger the successive computational steps of the network. Each spike of the *tic* cell induces a subsequent spike of *u*_*ϵ*_ one time step later. At this moment, the network tries to simulate an *ϵ*-transition of the counter machine. If such a transition is possible, the network performs it: this is the case at time steps *t* = 31, 181. Otherwise, the input encoding module retrieves the next input bit to be processed, and activates the corresponding cell *u*_0_ or *u*_1_ (blue pattern): this is the case at time steps *t* = 71, 101, 131, 161, 221, 251. In Fig 15 (cells *u*_0_, *u*_1_, *u*_*ϵ*_), we can see that on this input stream, the network processes the sequence of input symbols *ϵ*0011*ϵ*00.

Every time the network receives an input symbol (*ϵ*, 0 or 1), it simulates one transition of the counter machine associated to this input. The successive computational states of the machine are encoded into the successive values taken by *w*_*s*_(*t*) (cf. Fig 15, red curve in the upper graph). The changes in these synaptic weights are induced by the spiking patterns of cells *pre*_*s*_ and *post*_*s*_ (red patterns). The successive counter states of the machine, i.e., ‘zero’ or ‘non-zero’, are given by the activations of cells ‘= 0’ or ‘≠0’ of the counter modules, respectively (black patterns). The consecutive counter operations are given by the activations of cells *push*, *pop* and *test* (black patterns). The successive counter values of the machine are encoded into the successive values taken by $w_{c_{1}}\left(t\right)$ and $w_{c_{2}}\left(t\right)$ (orange curves of the upper graph). The changes in these synaptic weights are induced by the spiking pattern of cells $pre_{c_{j}}$ and $post_{c_{j}}$, for *j* = 1, 2 (orange patterns). The pics along these curves are caused by the testing procedures which increment and decrement back the values of the synapses without finally modifying their current values (cf. description of the counter module).

The computation of the network over input stream $u_{w_{1}}$ can be described by the successive synaptic weights $\left(w_{s}\left(t\right),w_{c_{1}}\left(t\right),w_{c_{2}}\left(t\right)\right)$ at time steps *t* = 30*k*, for 1 ≤ *k* ≤ 10. In this case, one has

$$\left(\begin{array}{c} w_{s}(t)\\ w_{c_{1}}(t)\\ w_{c_{2}}(t) \end{array}\right)=\left(\begin{array}{c} 0.1\\ 0.0\\ 0.0 \end{array}\right)\left(\begin{array}{c} 0.3\\ 0.0\\ 0.0 \end{array}\right)\left(\begin{array}{c} 0.3\\ 0.5\\ 0.5 \end{array}\right)\left(\begin{array}{c} 0.3\\ 0.75\\ 0.75 \end{array}\right)\left(\begin{array}{c} 0.6\\ 0.75\\ 0.5 \end{array}\right)\left(\begin{array}{c} 0.6\\ 0.75\\ 0.0 \end{array}\right)\left(\begin{array}{c} 0.4\\ 0.75\\ 0.0 \end{array}\right)\left(\begin{array}{c} 0.4\\ 0.5\\ 0.0 \end{array}\right)\left(\begin{array}{c} 0.4\\ 0.0\\ 0.0 \end{array}\right)\left(\begin{array}{c} 0.5\\ 0.0\\ 0.0 \end{array}\right).$$

Recall that state *n* and counter value *x* of $C_{k}$ are encoded by the synaptic weights *w*_*s*_(*t*) = *a*_*min*_ + *n* ⋅ *η* and *w*_*c*_(*t*) = *r*_*x*_ in N, respectively. Accordingly, the previous values correspond to the encodings of the following states and counter values (*q*, *c*_1_, *c*_2_) of the counter machine:

$$\left(\begin{array}{c} q\\ c_{1}\\ c_{2} \end{array}\right)=\left(\begin{array}{c} 0\\ 0\\ 0 \end{array}\right)\left(\begin{array}{c} 2\\ 0\\ 0 \end{array}\right)\left(\begin{array}{c} 2\\ 1\\ 1 \end{array}\right)\left(\begin{array}{c} 2\\ 2\\ 2 \end{array}\right)\left(\begin{array}{c} 5\\ 2\\ 1 \end{array}\right)\left(\begin{array}{c} 5\\ 2\\ 0 \end{array}\right)\left(\begin{array}{c} 3\\ 2\\ 0 \end{array}\right)\left(\begin{array}{c} 3\\ 1\\ 0 \end{array}\right)\left(\begin{array}{c} 3\\ 0\\ 0 \end{array}\right)\left(\begin{array}{c} 4\\ 0\\ 0 \end{array}\right).$$

These are the correct computational states and counter values encountered by the machine along the computation of input *w*_1_ = 001100 (cf. Fig 4). Therefore, the network simulates the counter machine correctly. The fact that the computations of the machine and the network terminate in state 4 and with *w*_*s*_(*t*) = 0.5 = 0.1 + 4 ⋅ *η*, respectively, means that inputs *w*_1_ and $u_{w_{1}}$ are accepted by both systems.

As another example, the computation of the network over the input stream

$$\begin{array}{c} u_{w_{2}}=0011101\text{end}\underset{\text{23}}{\underset{︸}{\emptyset \cdots \emptyset }}{\text{tic}}_{0}\underset{\text{29}}{\underset{︸}{\emptyset \cdots \emptyset }}{\text{tic}}_{1}\underset{\text{29}}{\underset{︸}{\emptyset \cdots \emptyset }}{\text{tic}}_{2}\cdots \end{array}$$

which corresponds to the encoding of *w*_2_ = 0011101, is displayed in Fig 16 (cells *u*_0_, *u*_1_, *u*_*ϵ*_). We see that on this input stream, the network processes the sequence of input symbols *ϵ*0011*ϵ*101. The successive synaptic weights $\left(w_{s}\left(t\right),w_{c_{1}}\left(t\right),w_{c_{2}}\left(t\right)\right)$ at time steps *t* = 30*k*, for 1 ≤ *k* ≤ 10 are

$$\left(\begin{array}{c} w_{s}(t)\\ w_{c_{1}}(t)\\ w_{c_{2}}(t) \end{array}\right)=\left(\begin{array}{c} 0.1\\ 0.0\\ 0.0 \end{array}\right)\left(\begin{array}{c} 0.3\\ 0.0\\ 0.0 \end{array}\right)\left(\begin{array}{c} 0.3\\ 0.5\\ 0.5 \end{array}\right)\left(\begin{array}{c} 0.3\\ 0.75\\ 0.75 \end{array}\right)\left(\begin{array}{c} 0.6\\ 0.75\\ 0.5 \end{array}\right)\left(\begin{array}{c} 0.6\\ 0.75\\ 0.0 \end{array}\right)\left(\begin{array}{c} 0.4\\ 0.75\\ 0.0 \end{array}\right)\left(\begin{array}{c} 0.2\\ 0.75\\ 0.0 \end{array}\right)\left(\begin{array}{c} 0.2\\ 0.75\\ 0.0 \end{array}\right)\left(\begin{array}{c} 0.2\\ 0.75\\ 0.0 \end{array}\right).$$

These values correspond to the encodings of the following states and counter values (*q*, *c*_1_, *c*_2_) of the counter machine:

$$\left(\begin{array}{c} q\\ c_{1}\\ c_{2} \end{array}\right)=\left(\begin{array}{c} 0\\ 0\\ 0 \end{array}\right)\left(\begin{array}{c} 2\\ 0\\ 0 \end{array}\right)\left(\begin{array}{c} 2\\ 1\\ 1 \end{array}\right)\left(\begin{array}{c} 2\\ 2\\ 2 \end{array}\right)\left(\begin{array}{c} 5\\ 2\\ 1 \end{array}\right)\left(\begin{array}{c} 5\\ 2\\ 0 \end{array}\right)\left(\begin{array}{c} 3\\ 2\\ 0 \end{array}\right)\left(\begin{array}{c} 1\\ 2\\ 0 \end{array}\right)\left(\begin{array}{c} 1\\ 2\\ 0 \end{array}\right)\left(\begin{array}{c} 1\\ 2\\ 0 \end{array}\right).$$

These are the correct computational states and counter values encountered by the machine working over input *w*_2_ = 0011101 (cf. Fig 4). Therefore, the network simulates the counter machine correctly. The fact that the computations of the machine and the network terminate in state 1 and with *w*_*s*_(*t*) = 0.2 = 0.1 + 1 ⋅ *η*, respectively, means that inputs *w*_1_ and $u_{w_{1}}$ are rejected by both systems.

## Discussion

We proposed a novel Turing complete paradigm of neural computation where the essential information is encoded into discrete synaptic levels rather than into spiking configurations, activation values or (attractor) dynamics of neurons. More specifically, we showed that *any* 2-counter machine—and thus any Turing machine—can be simulated by a recurrent neural network subjected to two kinds of spike-timing-dependent plasticity (STDP) mechanisms. The finitely many computational states and infinitely many counter values of the machine are encoded into finitely and infinitely many synaptic levels, respectively. The transitions between states and counter values are achieved via the two STDP rules. In short, the network operates as follows. First, the input stream is encoded and stored into the activation value of a specific analog neuron. Then, every time a *tic* input signal is received, the network tries to simulate an *ϵ*-transition of the machine. If such a transition is possible, the network simulates it. Otherwise, the network retrieves from its memory the next input bit to be processed, and simulates a regular transition associated with this input. These results have been illustrated by means of computer simulations. An STDP-based recurrent neural network simulating a specific 2-counter machine has been implemented and its dynamics analyzed.

We emphasize once again that the possibility to simulate *ϵ*-transitions is (unfortunately) necessary to the achievement of Turing completeness. Indeed, it is well-known that the class of *k*-counter machines that do not make use of *ϵ*-transitions is not Turing complete, for any *k* > 0. For instance, the language *L* = {*w*#*w*: *w* ∈ {0, 1}*} (the strings of bits separated by a symbol # whose prefix and suffix are the same), is recursively enumerable, but cannot be recognized by a *k*-counter machine without *ϵ*-transitions. The input encoding module, as intricate as it is, ensures the implementation of this feature. It encodes and stores the incoming input stream so as to be able to subsequently intersperse the successive regular transitions (associated to regular input symbols) with *ϵ*-transitions (associated to *ϵ* symbols). By contrast, a *k*-counter machine without *ϵ*-transitions could be simulated by an STDP-based neural network working in an online fashion. The successive input symbols would be processed as they arrive, and a regular transition be simulated for each successive symbol. An STDP-based neural net (as described in Fig 5) without input encoding module could simulate a *k*-counter machine without *ϵ*-transitions. One would just need to add sufficiently many delay layers to its input transmission module in order to have enough time to emulate each regular transition.

In the present context, the STDP-based RNNs are capable of simulating Turing machines working in the *accepting mode* (i.e., machines that provide accepting or rejecting decisions of their inputs by halting in an accepting or a rejecting state, respectively). But it would be possible to adapt the construction to simulate Turing machines working also in the *generative mode* (i.e., machines that write the successive words of a language on their output tape, in an enumerative way). To this end, we would need to simulate the program and work tape of M by an STDP-based RNN N (as described in Theorem 1), and the output tape of M by an additional neural circuit $N_{out}$ plugged to N. Broadly speaking, the simulation process could be achieved as follows:

- Every non-output move of M is simulated by the STDP-based RNN N in the usual way (cf. Theorem 1).
- Every time M is generating a new word *w* = *a*_1_⋯*a*_*n*_ on its output tape, use the circuit $N_{out}$ to build step by step the encoding ${\bar{r}}_{w}=\sum_{i=1}^{n}\frac{2a_{i}+1}{4^{i}}\in \left[0,1\right]$ of *w* and store this value in a designated neuron *c* (as described in the paragraph “Input encoding module”).
- When M has finished generating *w*, use the circuit $N_{out}$ to transfer the value ${\bar{r}}_{w}$ of *c* to another neuron *c*′, to set the activation value of *c* back to 0, and to output the successive bits of *w* by popping the the stack ${\bar{r}}_{w}$ stored in *c*′ (again, as described in the paragraph “Input encoding module”).

In this way, the STDP-based RNN N plugged to the circuit $N_{out}$ could work as a language generator: it outputs bit by bit the successive words of the language *L* generated by M. The implementation of the circuit $N_{out}$ is along the lines of what is described in the paragraph “input encoding module”.

Concerning the complexity issue, our model uses O(n) neurons and O(n) synapses to simulate a counter machine with *n* states. Moreover, the simulation works in real-time, since every computational step of the counter machine can be simulated in a fixed amount of 17 + 3(*n* − 1) time steps (17 time steps to transmit the next input bit up to the end of the detection modules, and at most 3(*n* − 1) time steps to perform the state and counter updates). In the context of rational-weighted sigmoidal neural networks, the seminal result from Siegelmann and Sontag uses 886 Boolean and analog neurons to simulate a universal Turing machine [4]. Recent results show that Turing completeness can be achieved with a minimum of 3 analog neurons only, the other ones being Boolean [58]. As for spiking neural P systems, Turing universality can be achieved with 3 or 4 neurons only, but this comes at the price of exponential time and space overheads (see [59], Table 1). In our case, the complexity of Turing universality is expected to be investigated in detail in a future work.

Regarding synaptic-based computation, a somehow related approach has already been pursued in the P system framework with the consideration of *spiking neural P systems with rules on synapses* [60]. In this case, synapses are considered as computational units triggering exchanges of spikes between neurons. The proposed model is shown to be Turing universal. It is claimed that “placing the spiking and forgetting rules on synapses proves to be a powerful feature, both simpler proofs and smaller universal systems are obtained in comparison with the case when the rules are placed in the neurons” [60]. In this context however, the information remains encoded into the number of spikes hold by the neurons, referred to as the “configuration” of the system. By contrast, in our framework, the essential information—the computational states and counter values—is encoded into discrete synaptic levels, and their updates achieved via synaptic plasticity rules.

As already mentioned, it has been argued that in biological neural networks “synapses change their strength by jumping between discrete mechanistic states rather than by simply moving up and down in a continuum of efficacy” [56]. These considerations represent “a new paradigm for understanding the mechanistic underpinnings of synaptic plasticity, and perhaps also the roles of such plasticity in higher brain functions” [56]. In addition, “much work remains to be done to define and understand the mechanisms and roles these states play” [56]. In our framework, the computational states and counter values of the machine are encoded into discrete synaptic states. However, the input stream to be processed is still encoded into the activation value of a specific analog neuron. It would be interesting to develop a paradigm where this feature also is encoded into synapses. Moreover, it would be interesting to extend the proposed paradigm of computation to the consideration of more biological STDP rules.

It is worth noting that synaptic-based and neuron-based computational paradigms are not opposite conceptions, but intertwined processes instead. Indeed, changes in synaptic states are achieved via the elicitation of specific neuronal spiking patterns (which modify the synaptic strengths via STDP). The main difference between these two conceptions is whether the essential information is encoded and memorized into synaptic states or into spiking configurations, activation values or (attractor) dynamics of neurons.

In biology, real brain circuits do certainly not operate by simulating abstract finite state machines. And with our work, we do intend to argue in this sense. Rather, our intention is to show that a bio-inspired Turing complete paradigm of abstract neural computation—centered on the concept of synaptic plasticity—is not only theoretically possible, but also potentially exploitable. The idea of representing and storing essential information into discrete synaptic levels is, we believe, novel and worthy of consideration. It represents a paradigm shift in the field of neural computation.

Finally, the impacts of the proposed approach are twofold. From a practical perspective, contemporary developments in neuromorphic computing provide the possibility to implement neurobiological architectures on very-large-scale integration (VLSI) systems, with the aim of mimicking neuronal circuits present in the nervous system [61, 62]. The implementation of our model on VLSI technologies would lead to the realization of new kinds of analog neuronal computers. The computational and learning capabilities of these neural systems could then be studied directly from the hardware point of view. And the integrated circuits implementing our networks might be suitable for specific applications. Besides, from a Machine Learning (ML) perspective, just as the dynamics of biological neural nets inspired neuronal-based learning algorithms, in this case also, the STDP-based recurrent neural networks might eventually lead to the development of new ML algorithms.

From a theoretical point of view, we hope that the study of neuro-inspired paradigms of abstract computation might contribute to the understanding of both biological and artificial intelligences. We believe that similarly to the foundational work from Turing, which played a crucial role in the practical realization of modern computers, further theoretical considerations about neural- and natural-based models of computation shall contribute to the emergence of novel computational technologies, and step by step, open the way to the next computational generation.

## Supporting information

S1 Files Python code. All python scripts generating the results of the paper are provided in an attached zip folder files.zip. The description of the different files is given in Read_me.txt.
(ZIP)
