# Supplementary figures and images for: Turing complete neural computation based on synaptic plasticity
Source: PLoS One. 2019 Oct 16;14(10):e0223451. doi: 10.1371/journal.pone.0223451 (PMC6795493; doi:10.1371/journal.pone.0223451)

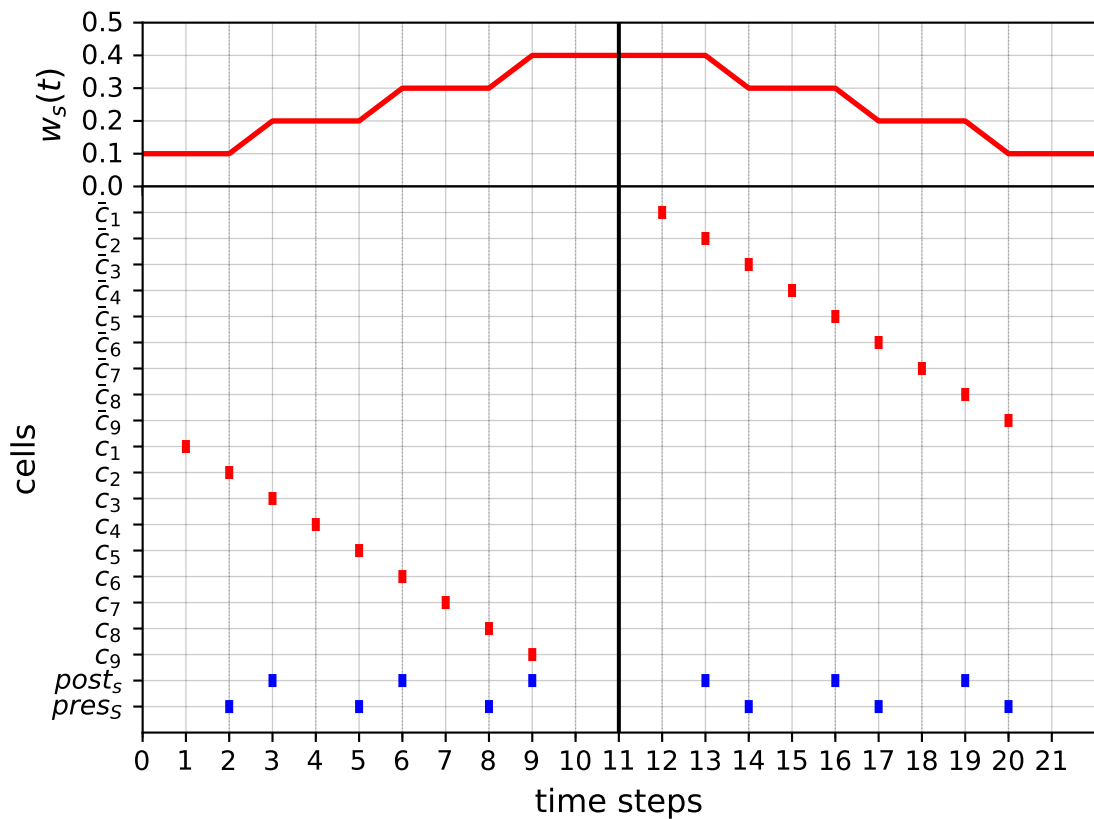

Supplement: S1 Files — All python scripts generating the results of the paper are provided in an attached zip folder files.zip. The description of the different files is given in Read_me.txt. (ZIP) [file pone.0223451.s001.zip › synaptic_computation/figures/raster_state.pdf]

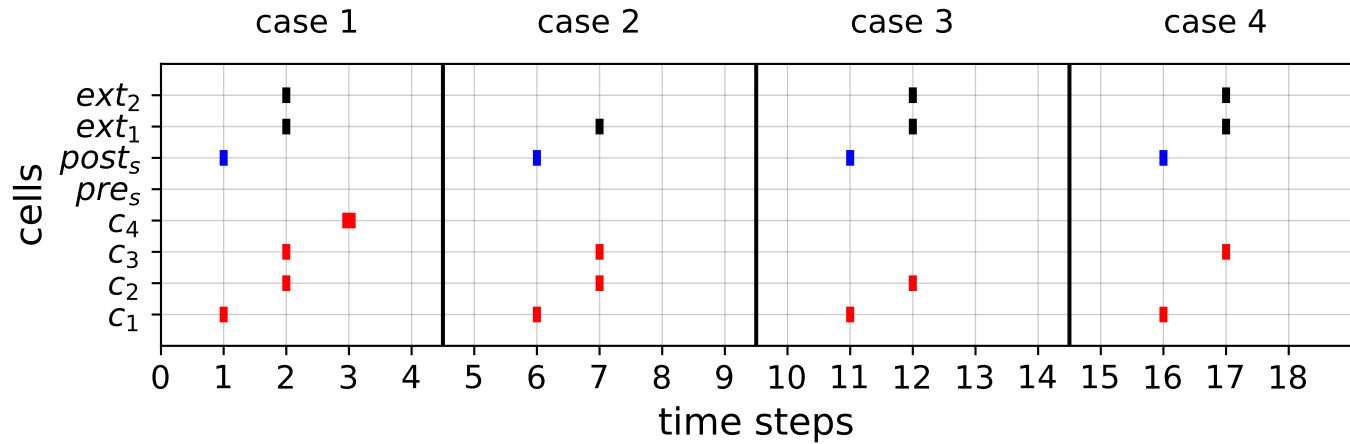

Supplement: S1 Files — All python scripts generating the results of the paper are provided in an attached zip folder files.zip. The description of the different files is given in Read_me.txt. (ZIP) [file pone.0223451.s001.zip › synaptic_computation/figures/raster_detection.pdf]

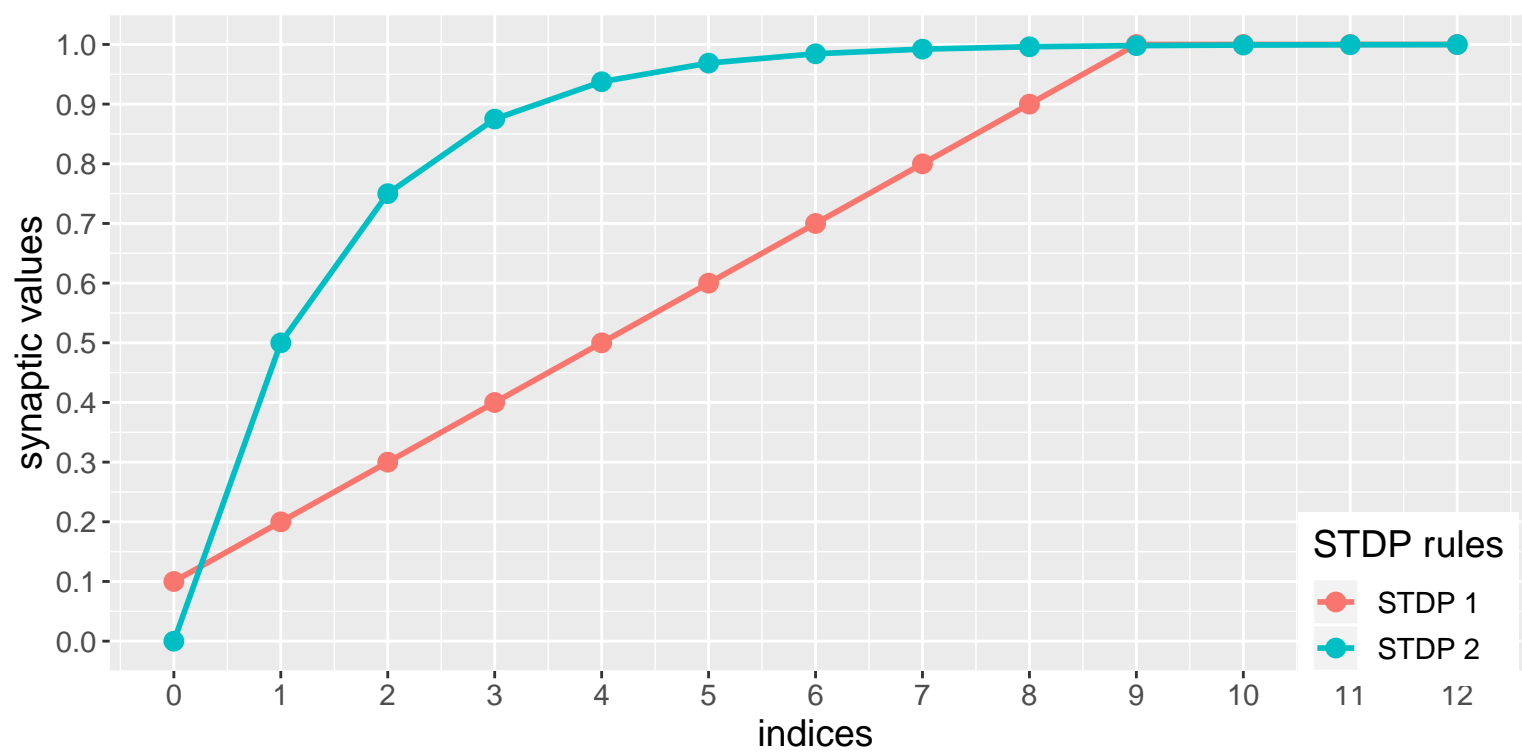

Supplement: S1 Files — All python scripts generating the results of the paper are provided in an attached zip folder files.zip. The description of the different files is given in Read_me.txt. (ZIP) [file pone.0223451.s001.zip › synaptic_computation/figures/STDP.pdf]

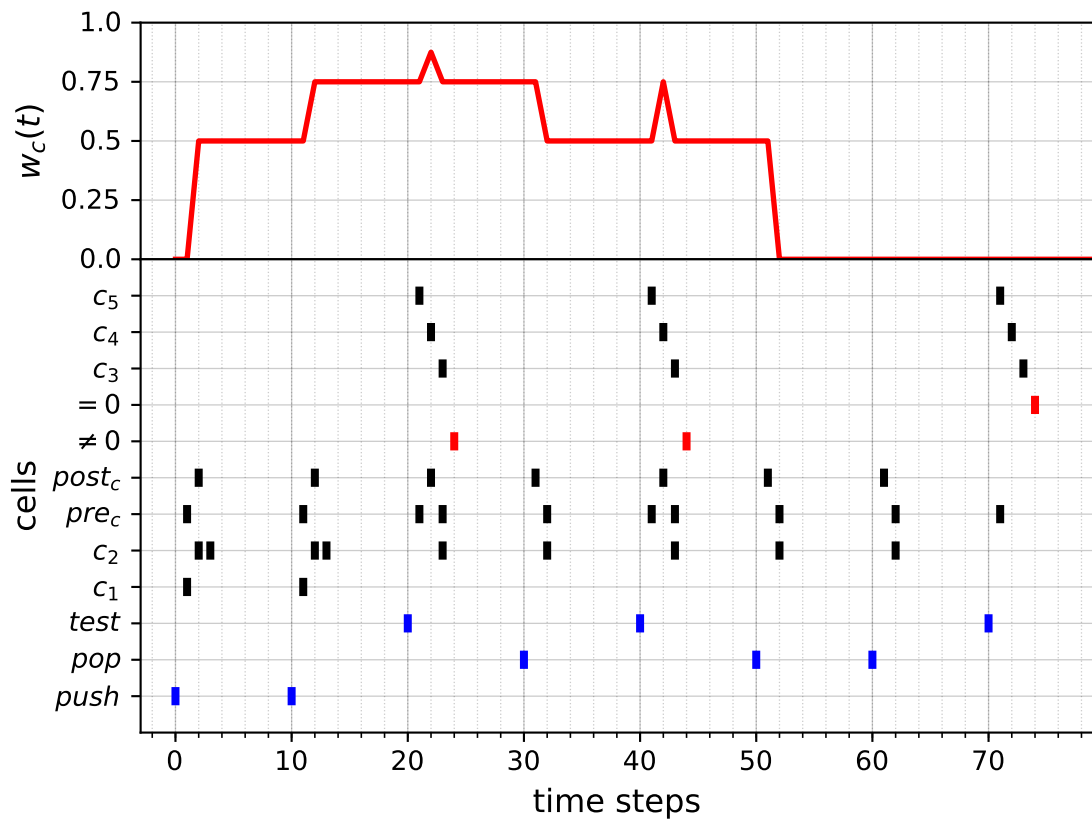

Supplement: S1 Files — All python scripts generating the results of the paper are provided in an attached zip folder files.zip. The description of the different files is given in Read_me.txt. (ZIP) [file pone.0223451.s001.zip › synaptic_computation/figures/raster_counter.pdf]

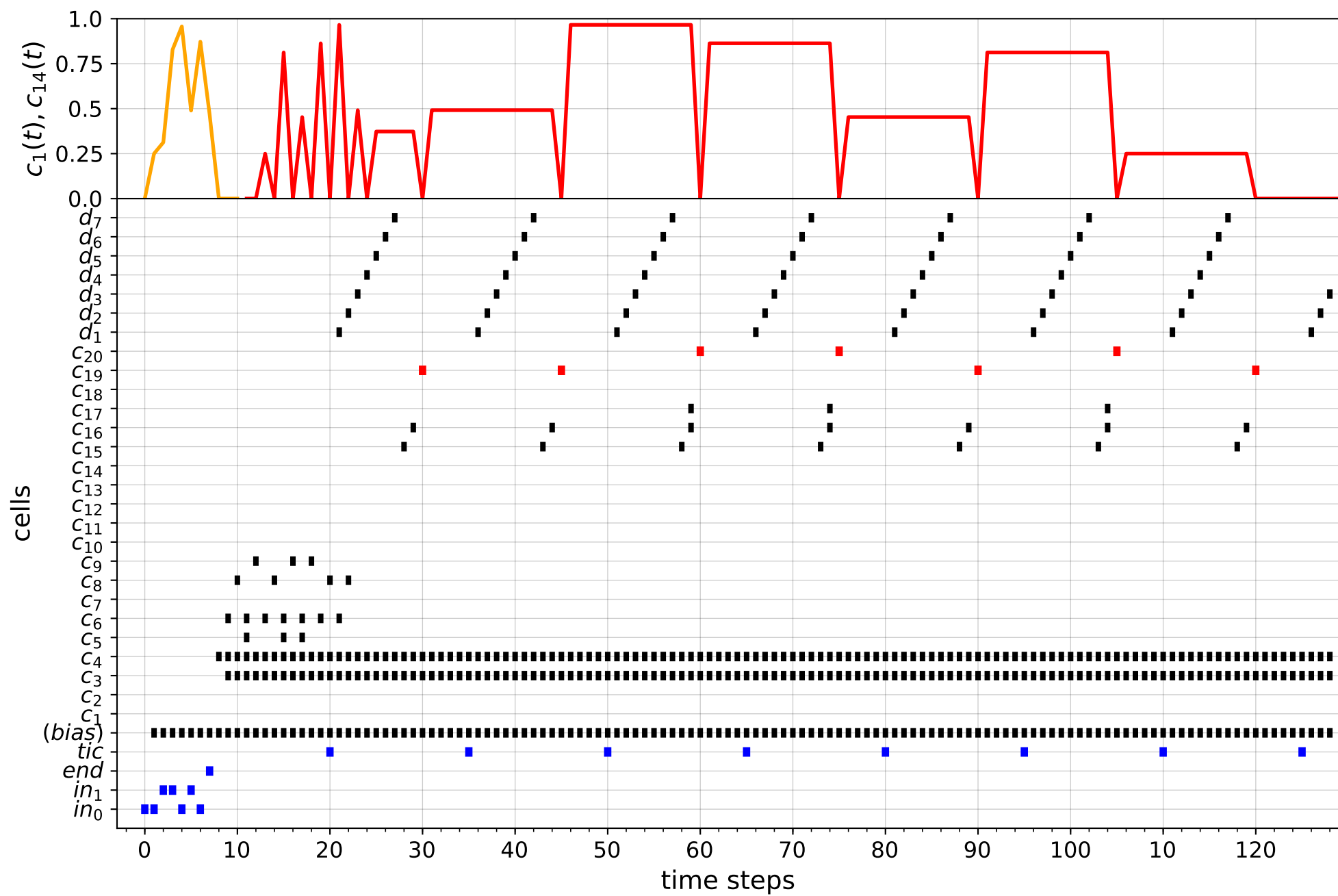

Supplement: S1 Files — All python scripts generating the results of the paper are provided in an attached zip folder files.zip. The description of the different files is given in Read_me.txt. (ZIP) [file pone.0223451.s001.zip › synaptic_computation/figures/raster_input_encoding.pdf]

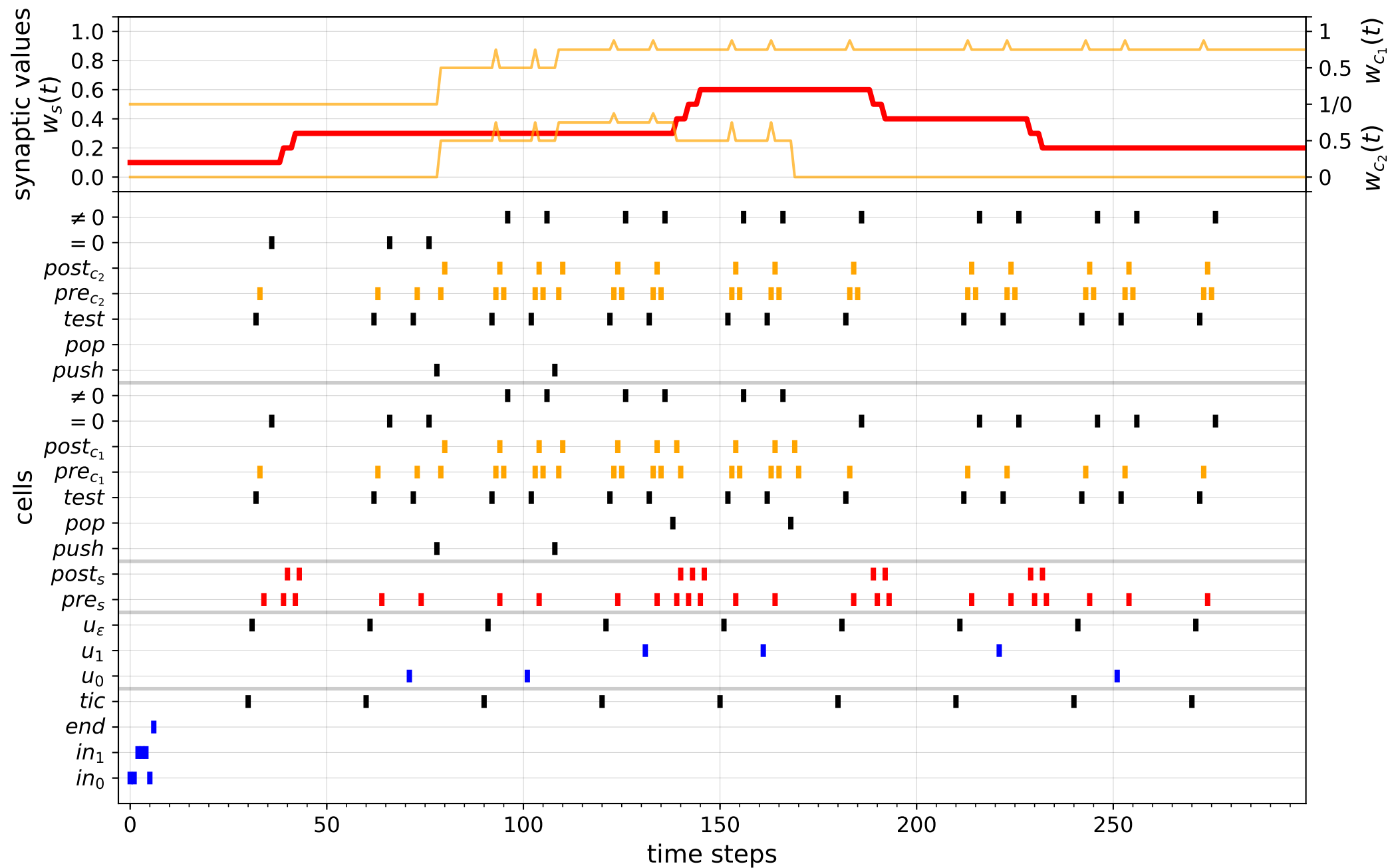

Supplement: S1 Files — All python scripts generating the results of the paper are provided in an attached zip folder files.zip. The description of the different files is given in Read_me.txt. (ZIP) [file pone.0223451.s001.zip › synaptic_computation/figures/simul2.pdf]

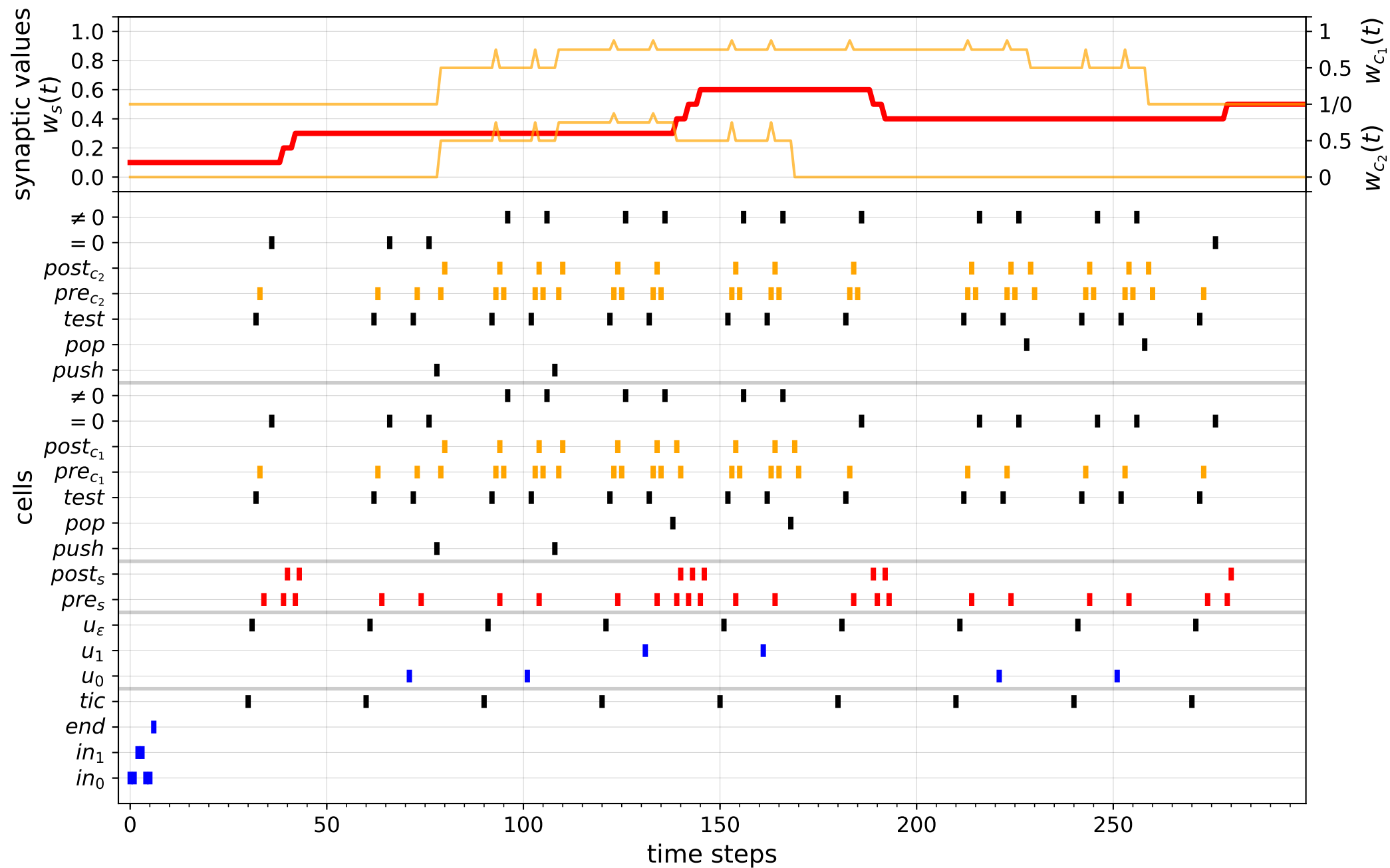

Supplement: S1 Files — All python scripts generating the results of the paper are provided in an attached zip folder files.zip. The description of the different files is given in Read_me.txt. (ZIP) [file pone.0223451.s001.zip › synaptic_computation/figures/simul1.pdf]
